# LILRB2/PirB mediates macrophage recruitment in fibrogenesis of nonalcoholic steatohepatitis

**DOI:** 10.1038/s41467-023-40183-3

**Published:** 2023-07-22

**Authors:** Dan-Pei Li, Li Huang, Ran-Ran Kan, Xiao-Yu Meng, Shu-Yun Wang, Hua-Jie Zou, Ya-Ming Guo, Pei-Qiong Luo, Li-Meng Pan, Yu-Xi Xiang, Bei-Bei Mao, Yu-Yu Xie, Zhi-Han Wang, Min Yang, Rui He, Yan Yang, Zhe-Long Liu, Jun-Hui Xie, De-Lin Ma, Ben-Ping Zhang, Shi-Ying Shao, Xi Chen, Si-Miao Xu, Wen-Tao He, Wen-Jun Li, Yong Chen, Xue-Feng Yu

**Affiliations:** 1 Division of Endocrinology, Department of Internal Medicine, Tongji Hospital, Tongji Medical College, Huazhong University of Science and Technology, Wuhan, China; 2 Branch of National Clinical Research Center for Metabolic Diseases, Hubei, China; 3 Computer Center, Tongji Hospital, Tongji Medical College, Huazhong University of Science and Technology, Wuhan, China

**Keywords:** Chronic inflammation, Chemokines

## Abstract

Inhibition of immunocyte infiltration and activation has been suggested to effectively ameliorate nonalcoholic steatohepatitis (NASH). Paired immunoglobulin-like receptor B (PirB) and its human ortholog receptor, leukocyte immunoglobulin-like receptor B (LILRB2), are immune-inhibitory receptors. However, their role in NASH pathogenesis is still unclear. Here, we demonstrate that PirB/LILRB2 regulates the migration of macrophages during NASH by binding with its ligand angiopoietin-like protein 8 (ANGPTL8). Hepatocyte-specific ANGPTL8 knockout reduces MDM infiltration and resolves lipid accumulation and fibrosis progression in the livers of NASH mice. In addition, PirB^−/−^ bone marrow (BM) chimeras abrogate ANGPTL8-induced MDM migration to the liver. And yet, PirB ectodomain protein could ameliorate NASH by sequestering ANGPTL8. Furthermore, LILRB2-ANGPTL8 binding-promoted MDM migration and inflammatory activation are also observed in human peripheral blood monocytes. Taken together, our findings reveal the role of PirB/LILRB2 in NASH pathogenesis and identify PirB/LILRB2-ANGPTL8 signaling as a potential target for the management or treatment of NASH.

## Abstract (web summary)

Inhibition of immunocyte infiltration and activation has been suggested to ameliorate hepatic inflammation and fibrosis in nonalcoholic steatohepatitis. Here, the authors show PirB/LILRB2 regulates the migration of macrophages during NASH by binding with ANGPTL8, which is involved in the regulation of NASH development.

## Introduction

Nonalcoholic fatty liver disease (NAFLD), which is one of the most common liver disorders, is associated with increased overall mortality. Nonalcoholic steatohepatitis (NASH), the inflammatory subtype of NAFLD, has a higher probability of progressing to end-stage liver diseases, such as cirrhosis and hepatic carcinoma^1,2^. However, there remain difficulties in the treatment of NASH due to a lack of approved pharmacological agents^3^. Therefore, it is urgent to identify novel targets for better implementation of NASH treatment.

Liver macrophages have been shown to be involved in the progression of steatohepatitis and subsequent hepatic fibrosis^4^. The liver harbors the largest proportion (~80%) of macrophages in the body^5^, and liver macrophages consist of heterogeneous populations, including Kupffer cells (KCs), which are resident and principally nonmigratory phagocytes with self-renewing ability, and monocyte-derived macrophages (MDMs), which are recruited from circulation. The macrophage pool of the liver can be rapidly expanded by infiltrated MDMs when KCs are decreased during diseases or after injury^6^. Experimental mouse models of NASH have displayed increased MDM infiltration and cytokine release, which are critical pathogenic events promoting steatohepatitis and hepatic fibrogenesis^7^. Hence, inhibition of MDM recruitment and activation is a widely accepted therapeutic strategy to attenuate steatohepatitis^7–10^.

Paired immunoglobulin-like receptor B (PirB) and its human ortholog receptor, leukocyte immunoglobulin-like receptor B-2 (LILRB2), are mainly expressed in various immunocytes and enable them to properly respond to extrinsic stimuli^11^. PirB/LILRB2 are classified as inhibitory receptors for their intracytoplasmic domain (ITIM motifs), which can recruit phosphatases SHP-1, SHP-2, or SHIP to negatively regulate cell activation^11^, such as downregulation of immune responses and inhibition of regeneration of adult neuron cells^12^. However, the role of PirB/LILRB2 in hepatic inflammation still requires to be clarified. Considering that the liver harbors many immunocytes and that the metabolic homeostasis of the whole body is largely maintained by hepatic immunologic balance, PirB/LILRB2, as an immune-inhibitory receptor, could play a pivotal role in metabolic diseases. By analyzing published RNA-sequencing data^13,14^, we found that LILRB2 expression in the liver is increased in NAFLD/NASH patients. As immunosuppression has been suggested to be effective for ameliorating hepatic inflammation and fibrosis in NASH^3^, we speculated that LILRB2/PirB, as immune-inhibitory receptors, would be potential targets for NASH therapy.

LILRB2 has been proven to be a receptor of several angiopoietin-like proteins (ANGPTLs), and the binding of ANGPTLs to these receptors supports the ex vivo expansion of hematopoietic stem cells^15^. In contrast to other ANGPTLs, ANGPTL8 is a cytokine that is exclusively expressed by the liver in humans^16^. More importantly, ANGPTL8 is associated with NAFLD both in mice and humans^17^, albeit with unsolved mechanisms. Recent studies have suggested that ANGPTL8 is a ligand for PirB^18,19^. Whether ANGPTL8 interacts with PirB and acts as a pro‐ or anti‐inflammatory factor and whether circulating ANGPTL8 exerts extracellular functions in metabolic diseases remain controversial^16,20^.

In this study, we identified that PirB/LILRB2 were expressed in hepatic macrophages and bound with their NASH-associated ligand (ANGPTL8) to trigger the recruitment of macrophages to the liver, providing evidence that the LILRB2/PirB-ANGPTL8 axis could be a pathogenic driver of NASH pathogenesis and fibrogenesis. In addition, an PirB/LILRB2-ANGPTL8 interaction facilitates the differentiation of hepatic macrophages to a proinflammatory phenotype by enhancing the phosphorylation of P38, AKT, and P65 signals, which in turn causes an aggravation in hepatocyte lipid accumulation and an exacerbation from simple hepatic steatosis to steatohepatitis. Our study provides insights into the role of PirB/LILRB2, as an immune-inhibitory receptor, in the regulation of hepatic inflammation and fibrogenesis. These observations also uncover a function of PirB/LILRB2 receptors in immunocyte migration and suggest them as potential targets for therapeutic intervention of NASH.

## Results

### Hepatic PirB/LILRB2 are increased in NASH patients and murine models

**Fig. 1:**
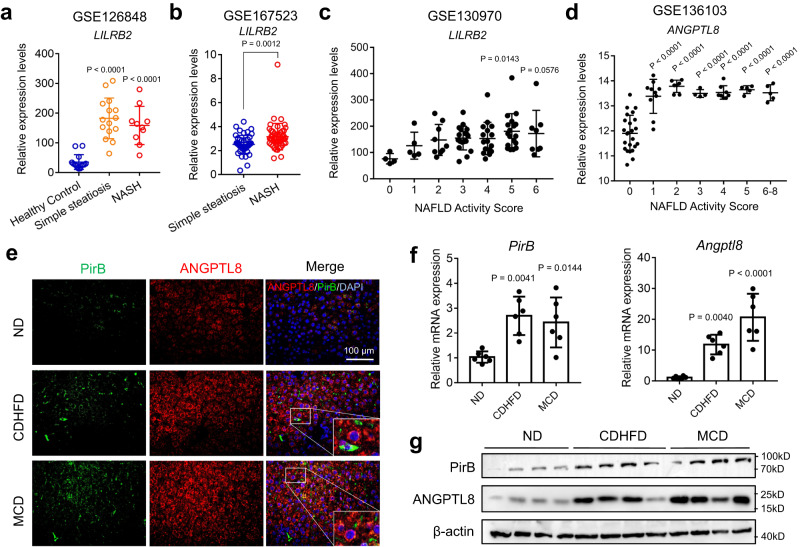
Increased ANGPTL8 and LILRB/PirB in the liver in human and murine NASH. **a**–**c** LILRB2 mRNA levels in the livers of NASH patients from GEO datasets (GSE126848, GSE167523, and GSE130970). **d** ANGPTL8 mRNA levels in the livers of NASH patients from GEO datasets (GSE136103). **e** Representative images showing immunofluorescence staining of PirB and ANGPTL8 in liver tissues. **f** Liver PirB and Angptl8 mRNA expression in 6-month CDHFD-fed and 2-month MCD-fed mice. **g** Western blot analysis of liver PirB and ANGPTL8 protein in 6-month CDHFD-fed and 2-month MCD-fed mice. Uncropped blots are provided in Source Data. Scale bar, 100 µm. All samples are biologically independent replicates and n indicates the number of biologically independent samples examined. Data shown are representative of four independent experiments with similar results (**e**, **g**). The data are shown as the mean ± s.e.m. (*n* = 6 mice/group) and were statistically analyzed by one-way ANOVA with Tukey’s multiple-comparison test (**a**, **c**, **d**, **f**) or two-tailed Student’s *t*-test (**b**). All the *p* values were two-sided and adjustments were made for multiple comparisons. kD relative molecular weight in kilodalton, ND normal diet, CDHFD choline-deficient high-fat diet, MCD methionine-choline deficient. Source data are available as a Source Data file.

To investigate hepatic expression of LILRB2 in patients during NASH progression, bioinformatic analysis was performed using RNA sequencing data published in the NCBI GEO repository^13,14^. Liver samples from NASH patients were histologically scored from 0 (normal control) to 8 according to the semiquantitative NASH-Clinical Research Network NAFLD Activity Score (NAS)^21^. LILRB2 mRNA expression was found to be increased in the livers of NAFLD patients (Fig. 1a–c) and was even higher in NASH patients (Fig. 1b, c). ANGPTLs have previously been demonstrated to be ligands for LILRB2^15^. Notably, unlike ANGPTL1-7, the expression of which was not changed during NASH (Supplementary Fig. 1a), ANGPTL8 was a unique secreted protein that increased in the full spectrum of NASH (NAS 1-8) (Fig. 1d).

To test whether PirB and its potential ligands were upregulated in mice during NASH progression, we developed murine NASH models by feeding C57BL/6 mice a 6-month choline-deficient high-fat diet (CDHFD) or a 2-month methionine-choline deficient (MCD) diet (Fig. 1e–g). NASH was determined by histological characterization of the liver (Supplementary Fig. 1b) and auxiliarily by remarkable increases in the liver-to-body weight ratio, hepatic TG content, and abnormalities in liver functions (Supplementary Fig. 1c). Compared with age-matched controls, livers of NASH models exhibited significantly elevated PirB and ANGPTL8 at both transcriptional and translational levels (Fig. 1e–g). However, ANGPTL1-7 levels were unchanged and significantly lower than ANGPTL8 levels in livers of the NASH model (Supplementary Fig. 1d, e). In addition, we noticed that ANGPTL8 was mainly expressed in liver (Supplementary Fig. 1f), whereas most of other ANGPTLs were expressed in different tissues indiscriminately^20^. Therefore, we considered ANGPTL8 protein as a hepatokine, which becomes an important ligand for hepatic PirB/LILRB2 and may be involved in NASH pathogenesis.

### Hepatic PirB is mainly expressed in monocyte-derived macrophages (MDMs)

**Fig. 2:**
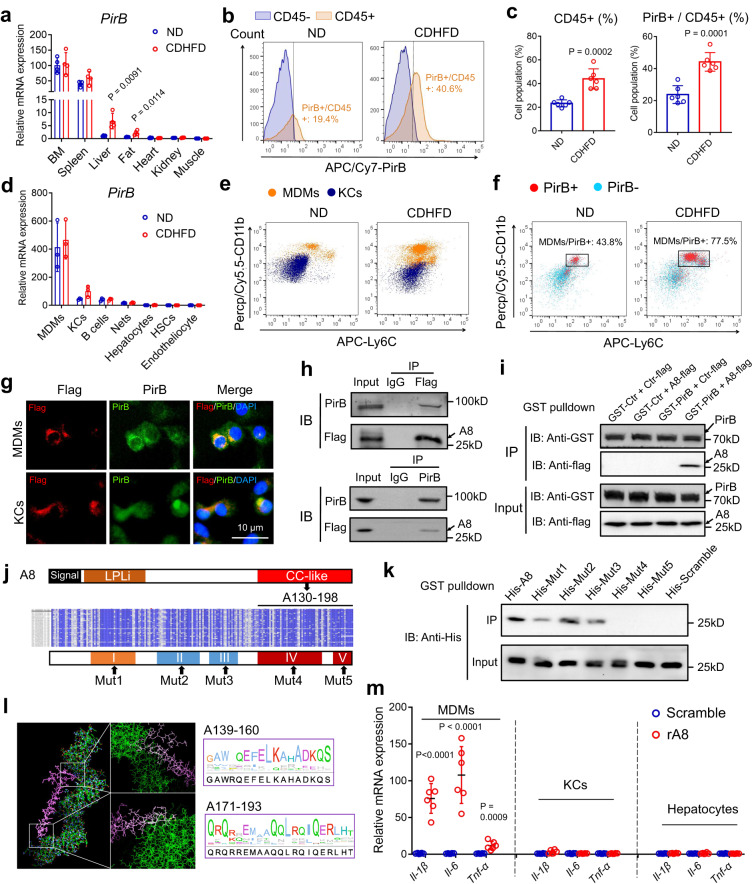
Hepatic PirB is mainly expressed in monocyte-derived macrophages (MDMs). **a** *PirB* mRNA expression in different tissues (*n* = 4). **b**, **c** Flow cytometry (FCM) and quantification of hepatic cells in the livers of the indicated mice (*n* = 6 mice/group). **d** *PirB* mRNA expression in different hepatic cells (*n* = 3). **e** Flow cytometry (FCM) of hepatic macrophages of the indicated groups. **f** Flow cytometry (FCM) of PirB^+^ cells of the livers. In the PirB^+^ CD45^+^ fraction, MDMs accounted for 43.8% (ND) and 77.5% (CDHFD). **g** Immunocytochemistry staining of Flag-targeted ANGPTL8 and PirB on MDMs and KCs treated with exogenous rA8. **h** Immunoprecipitation and western blot analyses showing the binding of flag-targeted A8 to PirB on MDMs. **i** In vitro GST pulldown assay to examine the interaction between ANGPTL8 and the PirB ectodomain. Scrambled PirB ectodomain and scrambled A8 were used as control (ctr). **j** Upper: Schematic of ANGPTL8 protein. LPLi: region inhibiting lipoprotein lipase (LPL) activity; Middle: amino acid sequences of ANGPTL8 in 77 mammalian species. The shades of blue indicate the degree of similarity, where dark blue indicates high similarity (16–55 and 130–198). Lower: Five mutant ANGPTL8 proteins (Mut1-5). **k** GST pulldown showing the interaction between mutant ANGPTL8 and the PirB ectodomain. **l** Binding models predicted by molecular docking and WebLogo plots of the potential binding region. **m** mRNA expression of cytokines in different hepatic cells treated with rA8 (*n* = 6). Scale bar, 10 µm. The data are shown as the mean ± s.e.m. and were statistically analyzed by two-tailed Student’s *t* test. All samples are biologically independent replicates and *n* indicates the number of biologically independent samples examined. Data shown are representative of three independent experiments with similar results (**g**–**I**, **k**). Uncropped blots are provided in Source Data. All the p values were two-sided. kD relative molecular weight in kilodalton, ND normal diet, CDHFD choline-deficient high-fat diet, MDMs monocyte-derived macrophages, KCs Kupffer cells, Nets neutrophil, HSCs hepatic stellate cells, rA8 recombinant ANGPTL8 protein, IP immunoprecipitation, IB immunoblotting, Mut mutant. Source data are available as a Source Data file.

PirB was mainly expressed in the immune and hematopoietic systems (bone marrow and spleen) in normal diet (ND)-fed mice (Fig. 2a). However, we noticed that the PirB distribution was changed under conditions of metabolic stress, as demonstrated by significantly increased hepatic PirB expression in CDHFD-fed mice (Fig. 2a). To verify the source of the increased hepatic PirB in NASH, we performed flow cytometry (FACS) and found that all the PirB^+^ cells in the livers were CD45^+^ (ND: 98.7% and CDHFD: 99.5%) (Fig. 2b). Considering that hepatocytes, hepatic stellate cells (HSCs), and endotheliocytes are CD45 negative^13^, hepatic PirB is most likely expressed in hepatic immunocytes (CD45^+^ population). In addition, we found that there were much more CD45^+^ cells in NASH livers than in healthy livers (Fig. 2b), and the proportion of PirB^+^ cells in CD45^+^ cells was significantly increased (from 19.4 to 40.6%) after a CDHFD (Fig. 2b, c), suggesting an accumulation of PirB^+^ hepatic immunocytes in NASH. Therefore, we examined the expression of *PirB* in different cell types in liver tissue and found that *PirB* was mainly expressed in hepatic macrophages, and no significant change in its mRNA levels was observed in NASH (after a CDHFD) (Fig. 2d).

Hepatic macrophages contain liver-resident Kupffer cells (KCs) and foreign monocyte-derived macrophages (MDMs), which infiltrate from circulation^22^. To discriminate KCs from MDMs, we first gated on KCs defined as CD11b^lo^ CLEC2^hi^ cells (Supplementary Fig. 2a). Among the remaining cells, we identified CLEC2^lo^ CD11b^hi^ CD64^+^ cells as MDMs (Supplementary Fig. 2a). It revealed that the hepatic MDM population was significantly increased in NASH (Fig. 2e), and the 77.5% PirB^+^ immunocytes in the NASH liver were MDMs (Fig. 2f; Supplementary Fig. 2b, c). In addition, 75% of CD45^+^ cells increased in NASH liver were PirB^+^ MDMs (Supplementary Fig. 2c, d). These data indicate that PirB^+^ MDMs contribute to the major population of macrophages in the NASH liver and their accumulation may play a significant role in NASH.

### PirB on MDMs binds to its ligand ANGPTL8

Due to a significant increase in hepatic PirB expression in NASH, we therefore explored the role of PirB in NASH liver. Given that PirB functions as a receptor, certain ligands should be essential for its regulation and possible intracellular signal delivery. In contrast to other potential ligands of PirB, we noticed that ANGPTL8 was a unique secreted protein that was mainly expressed in liver tissue (Supplementary Fig. 1f) and was associated with the NAFLD Activity Score (Fig. 1d), indicating its potential function in regulating hepatic PirB during NASH. To elucidate whether such a mechanism was at play in hepatic macrophages, we FACS-purified MDMs (F4/80^lo^ CD11b^hi^ CLEC2^lo^ Ly6c^+^) and KCs (F4/80^hi^ CD11b^lo^ CLEC2^hi^ Ly6c^−^) and found that PirB was highly expressed in MDMs and co-localized with its ligand ANGPTL8 on the cell membrane (Fig. 2g). Coimmunoprecipitation (Co-IP) assays further demonstrated that these two proteins displayed a physical binding (Fig. 2h; Supplementary Fig. 3a). A direct interaction between ANGPTL8 and the PirB ectodomain was further validated by GST pull-down assays (Fig. 2i). To verify their binding domain, we compared the amino acid sequences of ANGPTL8 in 77 mammalian species and identified two highly conserved regions: A16-55 and A130-198 (Supplementary Fig. 3b). Furthermore, ANGPTL8 contains a domain (corresponding to the conserved region A130-198), that is similar to but shorter than the CC domain of the other ANGPTLs and is thereafter referred to as the CC-like domain (Supplementary Fig. 3b). Next, we constructed plasmids containing the gene fragments, which can be encoded as truncated ANGPTL8 proteins (Supplementary Fig. 3c). Co-IP assays showed that truncated A130-198, A55-198, and full-length ANGPTL8 proteins were able to coimmunoprecipitated with pirB (Supplementary Fig. 3d), it indicated that A130-198 is a potential PirB-binding domain. According to the predicted binding sites, the mutant recombinant ANGPTL8 proteins with mutation in the I-V homologous regions were purified (Fig. 2j). GST-PirB pulldown assays revealed that the A139-160 (Mut4) and A171-193 (Mut5) of ANGPTL8 were responsible for its interaction with the PirB ectodomain (Fig. 2k). Molecular docking and WebLogo plots of amino acids were shown to describe the predicted binding regions (Fig. 2l). In addition, recombinant ANGPTL8 protein treatment significantly increased the inflammatory cytokine expression (such as *Il-1β*, *Il-6*, and *Tnf-α*) in MDMs, but not in KCs or hepatocytes, suggesting that ANGPTL8 exerts a proinflammatory role in MDMs but not in other hepatic cell types (Fig. 2m).

### ANGPTL8 promotes MDM migration and activation

**Fig. 3:**
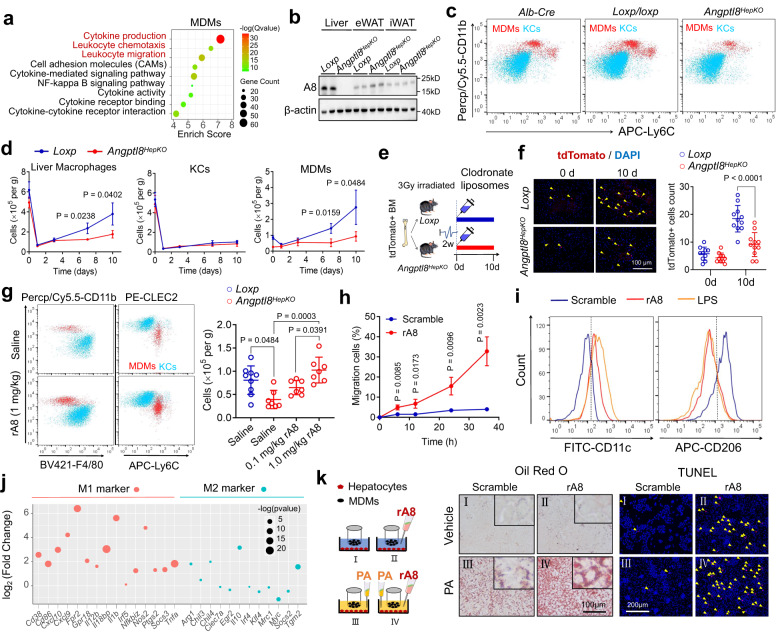
ANGPTL8 promotes MDM migration and activation. **a** Pathway enrichment analysis in MDMs treated with rA8. **b** Representative western blot for intracellular ANGPTL8 protein expression in different mouse tissues. Uncropped blots are provided in Source Data. **c** Flow cytometry analysis of KCs (CLEC2^hi^ F4/80^hi^ CD11b^lo^) and MDMs (CLEC2^lo^ CD11b^hi^ F4/80^lo^) in *Alb-Cre, loxp/loxp*, and *Angptl8*^*HepKO*^ mice. **d** Liver macrophage, KC, and MDM quantification in *loxp/loxp* and *Angptl8*^*HepKO*^ mice after KC deletion with clodronate liposomes (0, 1, 3, 7, 10 days) (*n* = 3 or 4 mice/group). **e** Experimental scheme to develop bone marrow (BM) chimeras using *loxp* and *Angptl8*^*HepKO*^ cells as recipients and congenic mTmG cells as donors (*n* = 10 or 11). **f** Representative images showing tdTomato+ cells in liver sections as well as quantification. **g** Flow cytometry analysis of KCs (CLEC2^hi^ F4/80^hi^ CD11b^lo^) and MDMs (CLEC2^lo^ CD11b^hi^ F4/80^lo^) in the livers of *Angptl8*^*HepKO*^ mice after rA8 injection (*n* = 7 or 9 mice/group). **h** Quantification of MDM migration to rA8 over time (*n* = 3 independent experiments). **i** Flow cytometry of MDM polarization after treatment with Scramble (blue), rA8 (red), and LPS (orange). **j** RNA sequence showing changes in M1/M2 markers of MDMs after rA8 stimulation. **k** Hepatocytes were cocultured with MDMs and treated with rA8 and PA, after which they were stained for lipid droplets (Oil Red O; scale bar, 100 µm) and apoptosis (TUNEL, highlighted by yellow arrow; scale bar, 200 µm). The data are shown as the mean ± s.e.m. and were statistically analyzed by one-way ANOVA with Tukey’s multiple-comparison test (**g**) or two-tailed Student’s *t*-test (**d**, **f**, **h**). All the *p* values were two-sided and adjustments were made for multiple comparisons. Data shown are representative of two (**b**) or four (**k**) independent experiments with similar results. *n* indicates the number of biologically independent samples examined. kD relative molecular weight in kilodalton, ND normal diet, CDHFD choline-deficient high-fat diet, eWAT epididymal white adipose tissue, iWAT inguinal white adipose tissue, rA8 recombinant ANGPTL8 protein, KCs Kupffer cells, MDMs monocyte-derived macrophages, PA palmitate. Source data are available as a Source Data file.

To identify the potential functions of ANGPTL8 in MDMs, we further analyzed ANGPTL8-induced changes in the transcriptional profile of MDMs through mRNA sequencing. Pathway enrichment analysis revealed that upregulated pathways in MDMs after ANGPTL8 treatment were correlated with leukocyte chemotaxis and migration (Fig. 3a). To clarify the source of intrahepatic ANGPTL8 protein, we examined ANGPTL8 expression in different hepatic cells (hepatocytes, MDMs, KCs, and HSCs), and found that ANGPTL8 was exclusively expressed in hepatocytes regardless of the nutrient conditions (ND or CDHFD) (Supplementary Fig. 4a). Therefore, we generated mice with hepatocyte-specific *Angptl8* knockout (hereafter referred to as *Angptl8*^*HepKO*^) to investigate the role of hepatocellular ANGPTL8 in the regulation of liver macrophages (Supplementary Fig. 4b). Specific deletion of ANGPTL8 in liver was determined by western blotting (Fig. 3b). In comparison to littermate controls (*Alb-cre* and *loxp/loxp*), the livers of *Angptl8*^*HepKO*^ mice exhibited a significantly decreased population in MDMs and Ly6C+ monocytes, but not in KCs (Fig. 3c; Supplementary Fig. 4c, d). Several previous studies have revealed that the depletion of KCs may result in compensatory generation of MDMs^23^. Nevertheless, in our study, KC depletion by clodronate liposomes (Supplementary Fig. 4e) led to an increase in MDM numbers only in the livers of control mice but not in those of *Angptl8*^*HepKO*^ mice (Fig. 3d). In addition, no significant proliferative difference between MDMs and KCs was detected after administration of ANGPTL8 in vitro (Supplementary Fig. 4f). Therefore, we speculated that *Angptl8*^*HepKO*^ mice exhibited a reduced capacity of MDM recruitment in liver, suggesting that ANGPTL8 may be required for MDMs to fill the empty niches that KCs vacated. This result was in agreement with the observed correlation between ANGPTL8 and leukocyte chemotaxis in pathway enrichment analysis (Fig. 3a).

To trace the origin of the increased MDMs after KC depletion, we first developed bone marrow (BM) chimeras by transplanting 5 × 10^6^ BM cells from congenic mTmG mice into *loxp/loxp* or *Angptl8*^*HepKO*^ recipient mice, which were exposed to low-dose irradiation (3 Gy) (Fig. 3e). Such a mild irradiation regimen would be sufficient to favor the engraftment of donor cells, which will then generate and enable the tdTomato+ monocytes to enter the bloodstream and spare radioresistant KCs according to previous studies^23^. Then, we examined the chimerism of circulating monocytes 2 weeks after BM transplantation and found that more than 70% of the Ly-6C^+^ monocytes (FACS-purified) were tdTomato^+^ (Supplementary Fig. 4g). Meanwhile, ~10% of the neutrophils and B cells and <2% of the T cells were tdTomato^+^ (Supplementary Fig. 4g). These chimeras were then injected with clodronate liposomes for 10 days. As expected, tdTomato+ cells were significantly increased in the livers of *loxp/loxp* mice, suggesting a recruitment of these cells from the circulation after KC depletion (Fig. 3f). However, a minor change in tdTomato^+^ cells was observed in the livers of *Angptl8*^*HepKO*^ mice (Fig. 3f). To validate the direct contribution of ANGPTL8 to the liver MDM pool, different amounts of recombinant ANGPTL8 protein were injected into *Angptl8*^*HepKO*^ mice through the tail vein. MDM levels were increased 4–6 h after rANGPTL8 injection in a dose-dependent manner (Fig. 3g), suggesting that the ANGPTL8-induced MDM migration would be earlier than ANGPTL8-related upregulation of chemokines (at least in 24 h). In vitro, MDMs were found to be transferred to the lower wells with recombinant ANGPTL8 protein treatment in a dose-dependent and time-responsive manner through a Transwell assay (Fig. 3h; Supplementary Fig. 4h).

Moreover, we analyzed the transcriptional changes in MDMs after ANGPTL8 treatment in vitro and found that M1 (inflammatory phenotype) marker genes were highly upregulated, whereas most M2 (anti-inflammatory phenotype) marker genes were downregulated or remained unchanged (Fig. 3j). Consequently, we referred to CD11c as an M1 marker and CD206 as an M2 marker with F4/80 co-staining to identify the polarization of MDMs by flow cytometry. After ANGPTL8 treatment, the CD11c^+^ population was increased (11.7% vs. 52.0%), whereas the CD206^+^ population was decreased (62.5% vs. 21.8%) relative to scrambled ANGPTL8 protein treatment (Fig. 3i). This MDM population shift was similar to the lipopolysaccharide (LPS)-induced shift (Fig. 3i). These results implied that ANGPTL8-treated MDMs bore more resemblance to M1-like macrophages.

### ANGPTL8-induced MDM activation promotes lipid accumulation in hepatocytes

Considering that M1 macrophages and their derived proinflammatory cytokines may regulate hepatocyte steatosis and apoptosis^24–26^, we therefore cocultured primary hepatocytes with MDMs to explore whether a crosstalk exists between these two cell types (Fig. 3k). ANGPTL8 treatment in the coculture resulted in a significant increase in the levels of certain proinflammatory cytokines, including IL-1β, IL-6, and TNF-α, in the culture medium (Supplementary Fig. 5a). Furthermore, we found that cytokines were significantly increased in ANGPTL8-treated MDMs but not hepatocytes (Supplementary Fig. 5b). In addition, hepatocytes that were cocultured with ANGPTL8-treated MDMs exhibited an aggravation in hepatocyte apoptosis, a promotion in palmitate (PA)-induced formation of intracellular lipid vacuoles and an increase in TG content (Fig. 3k; Supplementary Fig. 5c), and correspondingly, an upregulation in various lipogenic genes, including *Fasn*, *Srebp*, and *Scd-1* (Supplementary Fig. 5d). However, primary hepatocytes from either control or *Angptl8*^*HepKO*^ mice (without MDM coculture) did not show any difference in PA-induced lipogenesis (Supplementary Fig. 5e). These results indicated that macrophages were required for the effects of ANGPTL8 on lipid accumulation in hepatocytes.

### ANGPTL8 activates NF-κB signaling through PirB

**Fig. 4:**
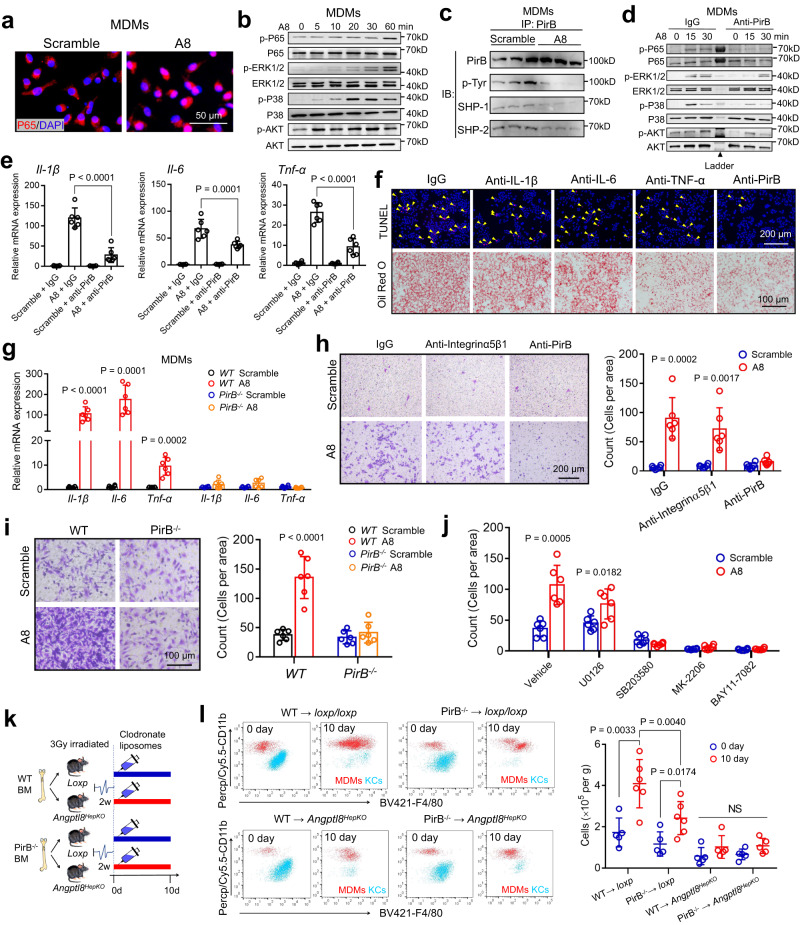
PirB mediates ANGPTL8-induced MDM migration and activation. **a** Nuclear translocation of P65 in MDMs. Scale bar, 50 µm. **b** Western blot analysis of the phosphorylation of signaling molecules in MDMs. **c** Assessment of the tyrosine phosphorylation of the PirB protein and interactions between PirB and SHP-1/2 by Co-IP assays. **d** Western blot analysis of the phosphorylation of signaling molecules in MDMs pretreated with indicated antibodies. **e** Effect of anti-PirB antibody on cytokine expression (*n* = 12 cells examined over four independent experiments). **f** Effect of neutralizing antibodies against cytokines and PirB on lipogenesis and apoptosis of hepatocytes cocultured with MDMs in the presence of rA8 and PA (*n* = 4 independent experiments). **g** Cytokine expression of MDMs from WT or PirB^−/−^ mice after rA8 or scrambled protein treatment (*n* = 6 cells examined over three independent experiments). **h** Effect of anti-PirB antibody and anti-integrin-α5β1 antibody on ANGPTL8-induced MDM migration (*n* = 6 cells examined over three independent experiments). Scale bar, 200 µm. **i** PirB^−/−^ MDMs were resistant to ANGPTL8-induced migration (*n* = 6 independent experiments). **j** Migration of MDMs pretreated with phosphorylation inhibitors before rA8 or scrambled protein stimulation (*n* = 6 cells examined over three independent experiments). U0126, p-ERK1/2 inhibitor; SB203580, p-P38 inhibitor; MK-2206, p-AKT inhibitor; BAY11-7082, p-P65 inhibitor. **k** Experimental scheme to develop BM chimeras. **l** Flow cytometry analysis and quantification of KCs (CLEC2^hi^F4/80^hi^CD11b^lo^) and MDMs (CLEC2^lo^CD11b^hi^F4/80^lo^) in the livers of the indicated BM chimeras (*n* = 5 or 6). The data are shown as the mean ± s.e.m. and were statistically analyzed by one-way ANOVA with Tukey’s multiple-comparison test (**e**, **l**) or two-tailed Student’s *t*-test (**g**, **h**, **i**, **j**). Data shown are representative of three independent experiments with similar results (**a**–**d**). Uncropped blots are provided in Source Data. All the *p* values were two-sided and adjustments were made for multiple comparisons. kD relative molecular weight in kilodalton, rA8 (or A8) recombinant ANGPTL8 protein, KCs Kupffer cells, MDMs monocyte-derived macrophages, PA palmitate, BM bone marrow, NS nonsignificant. Source data are available as a Source Data file.

Pathway enrichment analysis revealed that the NF-κB signaling pathway was upregulated after ANGPTL8 stimulation (Fig. 3a). Given that NF-κB is a key transcription factor implicated in the inflammatory signaling cascade of macrophages, we examined the changes of NF-κB signaling after ANGPTL8 stimulation and found that ANGPTL8 activated NF-κB in MDMs by translocating its P65 subunit to the nucleus (Fig. 4a). Consistently, an increase in the phosphorylation of the P65 subunit of NF-κB was observed after ANGPTL8 treatment in a time-dependent manner in MDMs (Fig. 4b). To elucidate the downstream regulatory mechanisms through which ANGPTL8 enhances NF-κB activation, we tested the phosphorylation levels of inflammation-associated signaling molecules (including JAK1-STAT1, STAT6, P38, ERK1/2, AKT, IRAK-1, and TAK-1) and found that ERK1/2, P38, and AKT phosphorylation in MDMs was enhanced after ANGPTL8 treatment (Fig. 4b). Since PirB harbors an intracellular ITIM motif that inhibits the phosphorylation of downstream signals when its tyrosine is phosphorylated^11^, we further tested the phosphorylation of ITIM in MDMs and found that ANGPTL8 treatment led to a reduction in ITIM tyrosine phosphorylation, accompanied by a decrease in SHP-1/2 recruitment (Fig. 4c). To obtain further insights into the role of PirB in this process, a neutralizing antibody against PirB ectodomains was used to block ANGPTL8 binding to MDMs (Supplementary Fig. 6a), and it revealed that the PirB antibody also abrogated ANGPTL8-induced phosphorylation of P65, ERK1/2, P38, and AKT in MDMs (Fig. 4d). These data suggested that ANGPTL8 decreased the autophosphorylation of PirB at tyrosine sites, thus reduced the recruitment of SHP1/2 and the activation of downstream signals (ERK1/2, P38, and AKT).

### PirB mediates ANGPTL8-induced MDM migration and activation

To examine whether PirB mediates the effect of ANGPTL8 on MDM migration and activation, PirB neutralizing antibody and PirB knockdown mice were used. We first found that MDMs pretreated with PirB antibodies exhibited lower mRNA expression of certain proinflammatory cytokines (including *Il-1β*, *Il-6* and *Tnf-α*) after ANGPTL8 stimulation (Fig. 4e). Moreover, PirB-blocking MDMs in the co-culture system (hepatocytes cocultured with MDMs) protected the cocultured hepatocytes from lipid accumulation and apoptosis (Fig. 4f), meanwhile, blockade of TNF-α using corresponding neutralizing antibodies in co-culture medium exerted a similar effect as PirB blockade. However, blockades of IL-6 and IL-1β failed to inhibit lipid accumulation and apoptosis in hepatocytes (Fig. 4f). These results indicated that blocking the binding of ANGPTL8 to PirB abrogated the proinflammatory polarization of MDMs as well as the lipogenesis in hepatocytes. To further verify that, we FACS-purified MDMs from PirB^−/−^ mice. Although slightly higher in basal expression, the mRNA expression of cytokines (*Il-1β*, *Il-6*, and *Tnf-α*) in MDMs from PirB^−/−^ mice showed no significant change after ANGPTL8 stimulation (unlike that in MDMs from the control mice) (Fig. 4g). Similar conclusions in cell migration were drawn when blocking or depleting PirB on MDMs (Fig. 4h, i). Blockade of PirB on MDMs significantly reduced ANGPTL8-induced migration of MDMs (Fig. 4h). Likewise, MDMs from PirB^−/−^ mice exhibited a lower response to ANGPTL8-induced migration relative to those from WT mice (Fig. 4i). ANGPTL2 has also been indicated to be associated with macrophage migration via integrin α5β1 in adipose tissues^27^. However, in our study, ANGPTL8-induced migration of macrophages was independent of integrin α5β1 receptors (Fig. 4h), possibly because of the absence of a fibrinogen-like domain (FLD, which is contained in the other seven ANGPTL members) in the ANGPTL8 protein structure (Supplementary Fig. 3b)^28^. Considering that the amino acid sequences of PirB ectodomains are highly homologous with those of PirA (over 92% identity) and that both receptors contain six extracellular immunoglobulin-like domains^11^, we also validated whether PirA contributes to ANGPTL8-induced MDM activation. Knockdown of PirA in RAW264.7 cells through lentiviral shRNA failed to decrease ANGPTL8-induced cytokine upregulation, whereas knockdown of PirB impaired the dose-dependent upregulation of *Il-1β*, *Il-6*, and *Tnf-α* by ANGPTL8 (Supplementary Fig. 6b–e), suggesting that PirB but not PirA mediates the above effects of ANGPTL8 on MDMs. Moreover, we observed that an inhibition in three main downstream signaling molecules of PirB (P38, AKT and NF-κB) through their corresponding inhibitors (SB203580, MK-2206, and BAY 11-7082) led to an abrogation in ANGPTL8-induced MDM activation (Supplementary Fig. 6f–h) and migration (Fig. 4j), whereas an ERK1/2 inhibitor (U0126) failed to exert similar effects.

To further understand PirB function in vivo, we generated BM chimeras by irradiating *loxp/loxp* and *Angptl8*^*HepKO*^ mice and reconstituted them with BM from WT or *PirB*^−/−^ mice, respectively (WT/*PirB*^−/−^→loxp/loxp; WT/PirB^−/−^→*Angptl8*^*HepKO*^) (Fig. 4k). Compared with the control (WT→*loxp/loxp*), MDM recruitment was significantly restrained in the liver of *loxp/loxp*-based *PirB*^−/−^ BM chimeras (PirB^−/−^→*loxp/loxp*) 10 days after clodronate liposome administration (Fig. 4l). In contrast, *Angptl8*^*HepKO*^-based *PirB*^−/−^ BM chimeras (PirB^−/−^→*Angptl8*^*HepKO*^) retained a similar MDM population as *Angptl8*^*HepKO*^-based WT BM chimeras (Fig. 4l). Taken together, our data showed that PirB mediates the effects of ANGPTL8 on MDM migration and activation both in vitro and in vivo.

### MDM depletion reduces inflammation and fibrosis in NASH

**Fig. 5:**
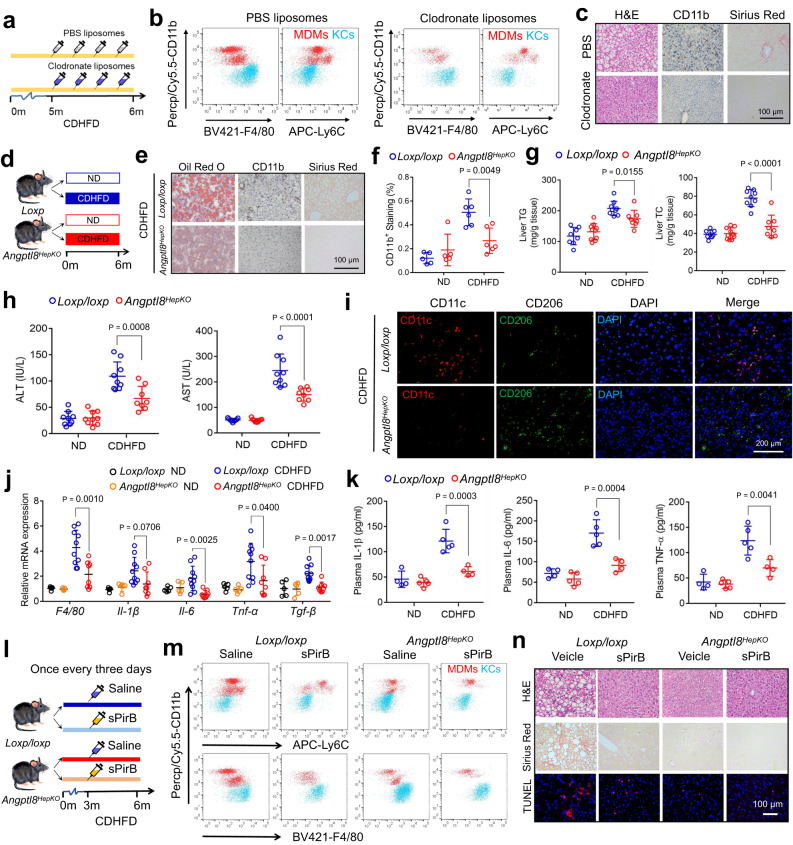
Hepatocyte-specific ANGPTL8 depletion and soluble PirB ectodomain (sPirB) protein ameliorate NASH. **a** Experimental setup for macrophage depletion in NASH livers. **b** Flow cytometry analysis of KCs (blue) and MDMs (red) after macrophage deletion. **c** Representative liver images of liver sections. Scale bar, 100 µm. **d** Experimental setup for inducing NASH in *Angptl8*^*HepKO*^ mice by CDHFD feeding. **e** Representative liver images of liver sections (scale bar, 100 µm). **f**–**h** Quantification of CD11b staining (**f**), liver TG and TC contents (**g**), and plasma ALT and AST levels (**h**) in the indicated groups (*n* = 8 or 10 mice/group). **i** Immunocytochemistry staining to identify M1 (CD11c) and M2 (CD206) macrophages in liver sections. Scale bar, 10 µm. **j** Hepatic inflammatory marker expression in the indicated groups (*n* = 5 mice for ND groups; 8 or 10 for CDHFD groups). **k** Plasma cytokine levels of the indicated mouse groups (*n* = 4 or 5 mice/group). **l** Experimental setup for sPirB injection. **m** Flow cytometry analysis of KCs (blue) and MDMs (red) 3 months after sPirB injection. *n* Representative liver images of liver sections. Scale bar, 100 µm. The data are shown as the mean ± s.e.m. and were statistically analyzed by one-way ANOVA with Tukey’s multiple-comparison test. All samples are biologically independent replicates and *n* indicates the number of biologically independent samples examined. Data shown are representative of three independent experiments with similar results (**c**, **n**). All the *p* values were two-sided and adjustments were made for multiple comparisons. MDMs monocyte-derived macrophages, KCs Kupffer cells, ND normal diet, CDHFD choline-deficient high-fat diet, sPirB soluble PirB ectodomain protein. Source data are available as a Source Data file.

Our previous data revealed that MDMs that accumulated in the liver of NASH mice are mainly originated from the bone marrow. To elucidate the role of MDMs in NASH, mice were fed a CDHFD for 6 months and received an injection of clodronate liposomes in the last month to deplete macrophages (Fig. 5a). Flow cytometry data showed that hepatic macrophages were significantly reduced after clodronate liposomes injection (Fig. 5b). As a result, macrophage depletion caused a decrease of 53% in lipid accumulation and a decrease of 72% in collagen fibers (Fig. 5c), concomitant with a 50% decrease in liver TG content and a 44.0% decrease in ALT plasma levels (Supplementary Fig. 7a, b). These results indicated that MDM depletion protects mice from the development of steatohepatitis.

### Hepatocyte-specific *Angptl8* knockout reduces MDM infiltration into the liver and ameliorates NASH

As Angptl8 promoted macrophage migration to the liver, we wondered whether a reduction in hepatic ANGPTL8 could inhibit the development of CDHFD-induced NASH. Considering that male mice were more stable when establishing the NASH model than female mice^29^, we opted to focus our study model mainly on male mice. Male *Angptl8*^*HepKO*^ mice together with control littermates were fed a CDHFD for 6 months (Fig. 5d). ANGPTL8 KO was determined by a dramatic decrease in hepatic expression of ANGPTL8 (Supplementary Fig. 7c). However, no statistically significant difference was observed in body weight (Supplementary Fig. 7e), food intake (Supplementary Fig. 7f), or body composition (Supplementary Fig. 7g) between *Angptl8*^*HepKO*^ mice and their control littermates when fed a ND or CDHFD. The liver weight and liver-to-body weight ratio of *Angptl8*^*HepKO*^ mice were significantly reduced compared to those of control littermates after 6 months challenging of CDHFD (Supplementary Fig. 7h). Importantly, specific *Angptl8* knockout in hepatocytes substantially attenuated MDM recruitment (CD11b staining and quantification in Fig. 5e, f), hepatic lipid accumulation (evidenced by Oil Red O staining in Fig. 5e and lipid content in liver in Fig. 5g), and the accumulation of extracellular matrix (by Sirius Red staining in Fig. 5e) in mouse liver. Considering that Cre transgenics might exhibit different phenotypes from mice with floxed sequences, we provided double controls (*loxp/loxp* and *Alb-cre*) to elucidate our findings in liver sections of *Angptl8*^*HepKO*^ mice (Supplementary Fig. 7d). *Angptl8*^*HepKO*^ mice exhibited lower plasma ALT and AST levels (Fig. 5h), an improved plasma lipid profile (Supplementary Fig. 7i), decreased M1 (CD11c) but increased M2 (CD206) macrophage numbers in comparison to the *loxp/loxp* mice (Fig. 5i). Moreover, *Angptl8* deficiency in hepatocytes attenuated the CDHFD-induced increase in the expression of hepatic inflammatory markers (including *Il-1β*, *Il-6*, *Tnf-α*, and *Tgf-β*) (Fig. 5j) and reduced relevant cytokine levels in the plasma (Fig. 5k). Considering that cytokines are closely related to insulin resistance, we also performed glucose tolerance tests (IPGTTs) and insulin tolerance tests (ITTs) and found that *Angptl8*^*HepKO*^ mice exhibited significantly alleviated insulin resistance (Supplementary Fig. 7j).

### Soluble PirB ectodomain (sPirB) attenuates NASH

Soluble ectodomains of receptors act as decoys to sequester endogenous ligands, which could result in a reduction in ligand binding and a subsequent attenuation in receptor signaling. Soluble PirB ectodomain protein (sPirB) has been thought to be a potential therapeutic approach for neurological diseases^30,31^. However, the effect of sPirB on NASH has not yet been reported. Thus, we generated sPirB containing the six immunoglobulin G (Ig)–like domains and His tags for its further purification and detection (Supplementary Fig. 7k). To determine the harboring ability of sPirB, the protein levels of sPirB in the liver were evaluated. 24 h after sPirB injection through the tail vein, there was an increased level of (His)_6_-positive proteins in the liver when the sPirB administration amount reached 1 mg/kg or 3.0 mg/kg (Supplementary Fig. 7l). The sPirB protein expression level was increased 12 h after 1 mg/kg sPirB injection, peaked at 24 h and persisted for 72 h (Supplementary Fig. 7l). Therefore, we injected CDHFD-fed control and *Angptl8*^*HepKO*^ mice with 1 mg/kg sPirB once every three days (Fig. 5l). Three months later, the fractions of MDMs in control mice, but not in *Angptl8*^*HepKO*^ mice, were significantly reduced (Fig. 5m). Moreover, sPirB administration resulted in a reduction in collagen fibers and hepatocyte apoptosis in the livers of the control mice (Fig. 5n), whereas no obvious improvement was observed in the livers of the *Angptl8*^*HepKO*^ mice (Fig. 5n). These results suggested that sPirB could be a potential therapeutic agent for NASH.

### LILRB2 mediates ANGPTL8-induced human peripheral blood monocyte migration

**Fig. 6:**
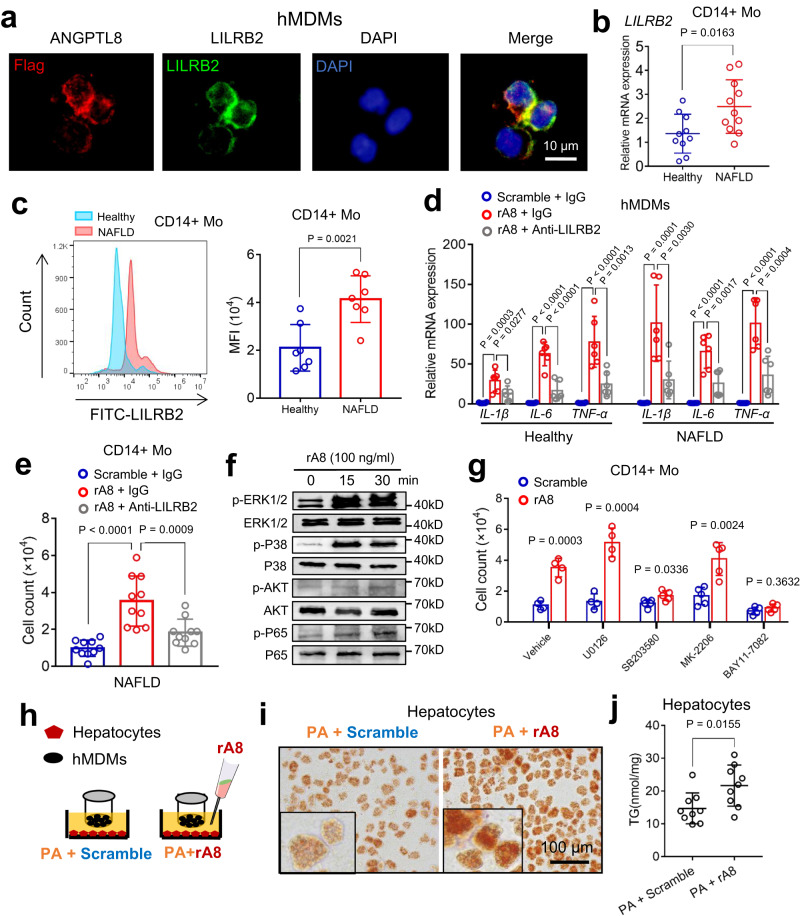
LILRB2 mediates ANGPTL8-induced human peripheral blood monocyte migration. **a** Immunocytochemistry staining of Flag-targeted ANGPTL8 and LILRB2 on human monocyte-derived macrophages (hMDMs) treated with exogenous rA8 (100 ng/ml). Scale bar, 10 µm. **b** LILRB2 mRNA expression in primary human peripheral blood monocytes from healthy adults and NASH patients (*n* = 10). **c** Flow cytometry analysis (left) of LILRB2 in the blood Mos (CD14+) from the indicated individuals. Mean fluorescence intensity (MFI) (right) of LILRB2 in blood Mos (CD14+) from the indicated individuals (*n* = 7). **d** Cytokine mRNA expression in hMDMs treated with scrambled protein and rA8 (n = 6). **e** Migration of CD14+ monocytes to ANGPTL8 were abrogated by the anti-LILRB2 antibody (*n* = 10). **f** Western blot analysis of the phosphorylation of signaling molecules downstream of LILRB2 in hMDMs treated with rA8. Uncropped blots are provided in Source Data. **g** Migration of CD14+ monocytes pretreated with phosphorylation inhibitors before rA8 or scrambled protein stimulation. U0126 p-ERK1/2 inhibitor, SB203580 p-P38 inhibitor, MK-2206, p-AKT inhibitor, BAY11-7082 p-P65 inhibitor (*n* = 4 or five independent experiments). (h) Experimental scheme to coculture hepatocytes with hMDMs. **i**, **j** Primary human hepatocytes were cocultured with monocyte-derived macrophages and treated with rA8 and PA, after which Oil Red O and TG (*n* = 9 biologically independent samples) were measured (scale bar, 100 µm). The data are shown as the mean ± s.e.m. and were statistically analyzed by two-tailed Student’s *t* test (**b,** **c**, **g**, **j**) and one-way ANOVA with Tukey’s multiple-comparison test (**d**, **e**). All samples are biologically independent replicates and n indicates the number of biologically independent samples examined (**b**–**e**). Data shown are representative of three independent experiments with similar results (**a**, **f**). All the *p* values were two-sided and adjustments were made for multiple comparisons. kD relative molecular weight in kilodalton, hMDMs human monocyte-derived macrophages, Mo monocytes, rA8 recombinant ANGPTL8 protein, MFI mean fluorescence intensity. Source data are available as a Source Data file.

To evaluate the effects of ANGPTL8 and LILRB2 on circulating monocytes in humans, we collected peripheral blood monocytes from healthy adults and NAFLD patients. We found that LILRB2 was highly expressed on human monocyte-derived macrophages (hMDMs), human monocytic THP1 cells, and human monocytic U937 cells, but was undetectable on human hepatic stellate cells (LX2), and an ANGPTL8 stimulation did not change the expression pattern of LILRB2 in all these cells (Supplementary Fig. 8a, b). Similar to the results observed in mice, we found that ANGPTL8 was also co-localized with LILRB2 on hMDMs (Fig. 6a). Interestingly, we noticed that monocytes from NAFLD patients showed higher LILRB2 expression than those from healthy individuals (Fig. 6b, c). We also identified a proinflammatory effect and migration-promoting effect of ANGPTL8 on hMDMs and CD14+ monocytes, as evidenced by the upregulation of cytokine mRNA expression and the increased cell migration after ANGPTL8 treatment in these cells (Fig. 6d, e). These promoting effects were abrogated by a neutralizing antibody against LILRB2 (Fig. 6d, e). Moreover, we observed the expression of the main downstream signaling molecules that we observed in mice (Fig. 6f) and found that ANGPTL8-induced increase in the phosphorylation of ERK1/2, P38, and P65 was similar to that was observed in mouse MDMs (Fig. 4b). Furthermore, the inhibition of P38 and P65 through their corresponding inhibitors (SB203580 and BAY 11-7082) led to an abrogation in ANGPTL8-induced migration (Fig. 6g), whereas an ERK1/2 inhibitor (U0126) and AKT inhibitor (MK-2206) failed to have a similar effect (Fig. 6g). Moreover, human hepatocyte lipogenesis was promoted when cocultured with ANGPTL8-treated hMDMs (Fig. 6h–j). Since HSCs also contribute to liver fibrosis, we also investigated the role of the ANGPTL8/LILRB2 axis in HSC-MDM crosstalk if exists (Supplementary Fig. 8c). In our study, we failed to observe the direct evidence supporting a crosstalk between ANGPTL8-activated macrophages and HSCs, since the fibrogenesis-related genes remained unchanged in HSCs when cocultured with macrophages in the presence of ANGPTL8 (Supplementary Fig. 8d, e).

## Discussion

**Fig. 7:**
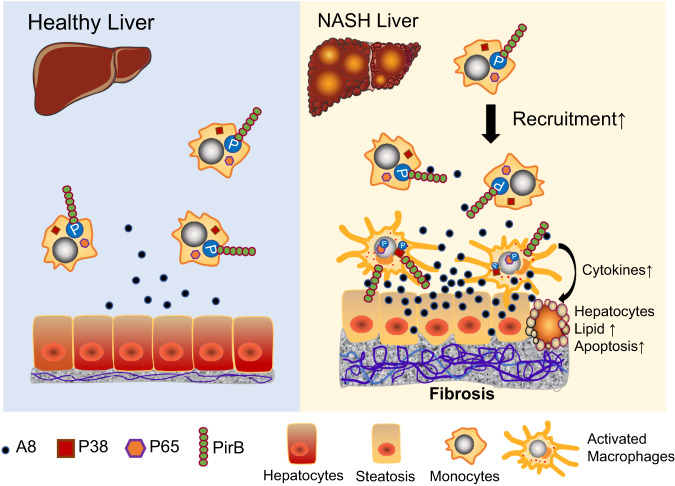
PirB/LILRB2 mediates macrophage recruitment to the liver and is involved in NASH pathogenesis and fibrogenesis. PirB/LILRB2-expressing circulating monocytes linger in the liver when they flow through the liver in response to a chemical concentration gradient of hepatic ANGPTL8. The pathologic elevation of ANGPTL8 during NASH accelerates the circulation of monocytes into the liver and promotes monocyte-macrophage transformation. The ANGPTL8-PirB/LILRB2-NF-κB axis induces the differentiation of mature macrophages into proinflammatory phenotypes in the liver and stimulates activated macrophages to produce more cytokines, which in turn causes aggravation of intracellular lipid accumulation and apoptosis of hepatocytes, finally resulting in the exacerbation of NASH fibrogenesis.

In our study, we identify PirB/LILRB2 on hepatic macrophages, which bind with their NASH-associated ligand (ANGPTL8) to trigger the recruitment of macrophages to the liver during NASH pathogenesis. In addition, the PirB/LILRB2-ANGPTL8 interaction induces the proinflammatory phenotype and stimulates cytokine production in the liver via activating the P38, AKT, and NF-κB signaling pathways, which in turn causes an aggravation in hepatocyte lipid accumulation and an exacerbation from simple steatosis to steatohepatitis (Fig. 7).

It has been long demonstrated that MDM recruitment is a critical pathogenic event that promotes steatohepatitis and subsequently hepatic fibrosis in NASH^4,7,8,32^. Although both MDMs and KCs accumulate in NASH liver^23,32^, hepatic inflammation during NASH is mainly induced by MDMs rather than resident KCs^32,33^. Besides, depletion of MDMs protects mice from diet-induced hepatic steatosis and ameliorates the progression of NASH^26,34,35^. Therefore, decline in MDM recruitment in liver could possibly be a potential strategy for NASH treatment. In our study, we found that liver-specific ablation of *Angptl8* reduced MDM recruitment and lipid accumulation and attenuates fibrosis in the liver of a murine NASH model. Thus, hepatic ANGPTL8 could be targetable to oppose inflammation and fibrosis in the liver.

In contrast to the other chemokines (such as CCL2, CCL3 and CCL5), which were previously reported to be associated with NASH^36–38^, ANGPTL8 is exclusively secreted from the liver in humans^39^. Since ~30% of the total blood volume of the human body passes through a network of hepatic sinusoids every minute^40^, our study suggests that circulating monocytes/macrophages may linger in the liver when they flow through hepatic sinuses in response to a chemical concentration gradient of high hepatic ANGPTL8 levels and then differentiate into mature macrophages. The pathologic elevation of ANGPTL8 in NASH would accelerate the infiltration of monocytes into the liver, promote monocyte-macrophage transformation, promote proinflammatory phenotypes and thus aggravate NASH progression.

To understand liver secreted ANGPTL8 exert its effects on MDMs, we focus on studying the receptors on MDMs. The results of the receptors fishing experiment reveals PirB as the most abundant receptor binding with ANGPTL8. Previous studies have already shown that ANGPTL8 physically interacts with PirB in hepatoma cells in mice^18^, and Zhang Z et al. have recently reported that ANGPTL8 accelerates liver fibrosis by binding with LILRB2 on HSCs^41^. Even though, we did not detect any LILRB2/PirB mRNA expression on CD45^−^ hepatic cells (hepatocytes, HSCs, and endotheliocytes). Instead, our subsequent experiments demonstrate that PirB receptors are mainly expressed on CD45^+^ immune cells (e.g., macrophages) in the liver. This conclusion is consistent with the well-accepted view that LILRB2/PirB are expressed on various hematopoietic cell lineages^11^.

More importantly, in our study, PirB^−/−^-BM chimeras (WT-based mice) exhibit decreased hepatic MDM infiltration, indicating LILRB2/PirB as new chemokine receptors on MDMs that mediate macrophage migration to the liver. Furthermore, administration of the soluble form of the PirB ectodomain protein restrains CDHFD-induced NASH, suggesting that the PirB ectodomain protein could be a potential therapeutic agent for NASH treatment by sequestering certain deleterious ligands. Simultaneously, the above observations raise another question: are there other hepatic ligands targeting PirB except for ANGPTL8, such as other ANGPTL family members, which have been demonstrated as LILRB2 ligands^15^. We find that ANGPTL1, 2, 5, and 7 are not expressed in the liver (Supplementary Fig. 1d) and ANGPTL3, 4, and 6 do not interact with LILRB2^15^. Albeit that, due to the fact that ANGPTL8 could form a complex with ANGPTL3^16^, there might be a possibility that ANGPTL3-ANGPTL8 complex provides an FLD domain, which endows ANGPTL8 to interact with the integrin (tightly related to cell adhesion) or function at a lower level.

In our study, ANGPTL8 does not facilitate the release of classical chemokines, such as MCP1 (CCL2), MIP-α (CCL3), and MIP-β (CCL4), which have been previously implicated in NAFLD/NASH^42^. Besides, classical chemokine receptors such as CCR1, CCR2, and CCR5 were not changed. Therefore, ANGPLT8/PirB-induced MDM migration would be independent of the classical chemotaxis pattern. Moreover, we also notice that some nonclassical chemokines with largely unknown regulatory mechanisms involved in monocyte recruitment were upregulated after long-term ANGPTL8 stimulation. Whether these chemokines are involved in the regulatory effects of ANGPTL8 on MDM migration is unclear. Our in vitro/vivo experiments demonstrate that an MDM migration occurs in 4–6 h after ANGPTL8 treatment, which is much earlier than the assemble and release of chemokines (as least 24 h). Therefore, we opt to consider this chemotaxis as a direct response to ANGPTL8-PirB axis. Subsequent studies are required to illuminate the mechanisms of other non-canonical migration.

Previous studies in NASH patients suggest that the accumulation and inflammatory polarization (M1-like) of hepatic macrophages is a hallmark feature of disease progression^43^, and M1-like macrophages have been shown to be involved in the mechanisms of hepatocyte lipid metabolism^44,45^. In our study, ANGPTL8 orients macrophages into an inflammatory phenotype, which increase lipid accumulation in the cocultured hepatocytes, elevate lipogenic gene expression, and aggravate apoptosis of these cells. These results are consistent with the increasing evidence supporting that ANGPTL8 functions as a proinflammatory factor^46–48^, and the observation that hepatocytes exhibit increased lipogenesis and apoptosis when cocultured with M1 macrophages in other studies^24–26^. We further show that the proinflammatory effects of ANGPTL8 in the liver are mainly regulated by its interaction with PirB, as evidenced by abolished cytokine expression in PirB^−/−^ macrophages or reduced cytokine expression in WT macrophages after treatment with neutralizing antibodies against PirB.

To understand the molecular mechanisms, it is necessary to elucidate the signaling pathways by which the ANGPTL8-PirB axis regulates cytokine production and MDM migration in the liver. Previous studies have shown that the activation of the transcription factor NF-κB is intensively involved in macrophage activation and is known to be central to the inflammatory aspects of NASH^11,18,49^. A previous study suggests that ANGPTL8 is a negative regulator of NF-κB^50^. In contrast, our study observes an increase in the translocation of the P65 subunit of NF-κB to the nucleus in MDMs stimulated with ANGPTL8. Furthermore, examination of the main upstream signaling pathways of NF-κB in MDMs shows that only ERK1/2, P38, and AKT robustly respond to ANGPTL8 stimulation. PirB blockade with neutralizing antibodies or PirB knockdown through lentiviral infection abrogates the effects of ANGPTL8-induced phosphorylation of ERK1/2, P38, and AKT, suggesting that ANGPTL8-responsive signals are downstreams of PirB. This result is consistent with previous studies showing that PirB regulates the phosphorylation of ERK1/2, P38, and AKT^11,18^.

Mice with conditional hepatic depletion of ANGPTL8 lack an important initiating chemokine to attract peripheral monocytes/macrophages into the liver. However, we notice that circulating ANGPTL8 in *Angptl8*^*HepKO*^ mice is not completely eliminated, especially under excess nutrient conditions, probably due to its secretion from adipose tissue^51,52^. In our study, body weight and adipose tissue weight are similar between *Angptl8*^*HepKO*^ and control mice, whereas *Angptl8*^*−/−*^ mice previously generated using VelociGene technology exhibit diminished accretion of adipose tissue^53^. This indicates that ANGPTL8 in adipose tissue is important for adipose expansion and that its function is independent of hepatic ANGPTL8. In addition, the lipid profile observed in *Angptl8*^*HepKO*^ mice is consistent with prior observations in *Angptl8*^*−/−*^ mice, implying that the improvement in blood lipids in *Angptl8*^*HepKO*^ mice primarily results from inhibition of ANGPTL8 in the liver rather than in the fat.

Nevertheless, we notice that there is discrepancy regarding the metabolic phenomenon of ANGPTL8 knockout mice, possibly due to the sex of the study groups^16,52–54^. Physiological levels of sex hormones vary significantly in premenopausal women, influencing the susceptibility of reproductive women to NAFLD^29^. In our previous study (the REACTION study), ANGPTL8 levels are significantly higher in men than in women at almost all age stages, especially in the ≤55-year-old population^55^. This data indicates that female hormones could affect ANGPTL8 levels, which is supported by a recent standpoint that estrogen inhibits ANGPTL8 expression in liver cells^56^. Although metabolic phenotype of *Angptl8*^*HepKO*^ is improved more significantly in male mice than in females, there is no significant difference between males and females regarding the improvement in hepatic inflammation and the reduction in macrophage recruitment and fibrogenesis.

In summary, ANGPTL8-PirB/LILRB2 is an important signaling axis for NASH pathogenesis via a crosstalk between hepatocytes and macrophages. It is possible that an increased ANGPTL8 level in the NASH liver results in an induction in hepatic macrophage polarization, an enhancement in proinflammatory cytokine production, and an increase in the recruitment of circulating monocytes/macrophages in the liver. Thus, blocking the binding of ANGPTL8 to PirB/LILRB2 receptors could be a promising therapeutic approach for the treatment of NASH. A better understanding of the biological function of the ANGPTL8-PirB/LILRB2 axis in inflammation would lead to a more profitable development of possible therapeutic agents for NASH treatment. Collectively, we reveal a previously unappreciated role of PirB/LILRB2 in NASH pathogenesis and identified ANGPTL8-PirB-NF-κB signaling as a potential target for the management of NASH in the future.

## Methods

### Animals

All animal protocols were approved by the Institutional Animal Care and Use Committee of the Institute of Model Animal of Tongji Hospital, Huazhong University of Science and Technology. The animals received humane care according to the criteria outlined in the Guide for the Care and Use of Laboratory Animals prepared by the National Academy of Sciences and published by the National Institutes of Health. Wild-type C57BL/6 J mice were obtained from Beijing Huafukang Bioscience Co., Ltd. (Beijing, China). Hepatocyte-specific *Angptl8*-knockout (*Angptl8*^*HepKO*^) mice were generated by Cyagen Biosciences Inc. (Guangzhou, China). Alb-cre mice, PirB knockout, and mTmG mice were purchased from Cyagen Biosciences Inc. (Guangzhou, China). The mice were given free access to food and water and housed in a temperature-controlled environment (23 ± 2 °C) with a 12/12-h light/dark cycle. NASH models were induced by feeding mice a choline-deficient high-fat diet (CDHFD, D05010403, Research Diets, 60 kcal% fat, 20 kcal% carbohydrate, and 20 kcal% protein, Medicience Ltd., Jiangsu, China) for 6 months, or a methionine-choline deficient (MCD, 21 kcal% fat, 63 kcal% carbohydrate, and 16 kcal% protein, Medicience Ltd., Jiangsu, China, Cat#MD12052) diet for 2 months (with the similar endpoint ages). Control mice were maintained with a normal diet (ND, 10 kcal% fat, 70 kcal% carbohydrate, and 20 kcal% protein, Jiangsu Xietong, Inc., Nanjing, China, Cat# SWS9102). During the experiments, body weight, food intake, fasting blood glucose and fasting serum, insulin concentration were assayed. Body composition was analyzed by NMR (Bruker SkyScan1276). To perform in vivo glucose tolerance tests (GTT) and insulin tolerance tests (ITT), mice were intraperitoneally injected with 1 g of glucose ((Sigma-Aldrich Co. St. Louis, MO, USA)) or 1 U insulin (Novolin R, Novo Nordisk Co., Bagsvaerd, Denmark)) per kg body weight, respectively. Mice were euthanized by carbon dioxide (CO_2_) asphyxiation inhalation and cervical dislocation was performed as secondary euthanasia procedure, and then the tissues were isolated.

### Liver macrophage depletion

Macrophage depletion was achieved by intraperitoneal injection of liposomes containing clodronate or PBS (5 mg/ml, 100 µl per 10 g mouse, Yeasen Biotechnology Co., Ltd., Shanghai, China). Mice with NASH received an intraperitoneal injection of clodronate liposomes once a week in the last month of the diet intervention (CDHFD).

### Total body irradiation and bone marrow transplantation

*Loxp* and *Angptl8*^*HepKO*^ mice were exposed to 3 Gy total body irradiation. Bone marrow cells were harvested from donor mice by gently flushing their femurs, and 5 × 10^6^ cells were intravenously injected into each recipient mouse. A two-week recovery period was observed to ensure donor bone marrow engraftment and blood monocyte reconstitution.

### Flow cytometry

Cell suspensions were stained with appropriate antibodies for 30 min on ice. The commercial antibodies used in this study included anti-mouse FITC-CD45 (BioLegend, CA, USA, #103108; 1 µg per 10^6^ cells), PE-CLEC2 (BioLegend, CA, USA, #146104; 1 µg per 10^6^ cells), BV421-F4/80 (BioLegend, CA, USA, #123137; 1 µg/10^6^ cells), Pe/Cy7-CD64 (BioLegend, CA, USA, #128016; 1 µg per 10^6^ cells), Percp/Cy5.5-CD11b (BioLegend, CA, USA, #101228; 1 µg per 10^6^ cells), APC/Cy7-PirB (R&D, CA, USA, #FAB2754S; 5 µg per 10^6^ cells), APC-Ly6C (BioLegend, CA, USA, #128016; 1 µg per 10^6^ cells), APC-Ly6G/Ly-6C (Gr-1) (BioLegend, CA, USA, #108411; 1 µg per 10^6^ cells), PerCP-CD19 (BioLegend, CA, USA, #115531; 1 µg per 10^6^ cells), APC-CD3 (BioLegend, CA, USA, #100235; 1 µg per 10^6^ cells), APC-CD206 (BioLegend, CA, USA, #141707; 2 µg per 10^6^ cells), and FITC-CD11c BioLegend, CA, USA, #117305; 1 µg per 10^6^ cells); and anti-human PE-CD14 (BD Biosciences Pharmingen, USA, #555398; 20 µl per 10^6^ cells), Mouse anti-human LILRB2 (R&D, R&D Systems, MN, USA, #MAB2078; 0.25 µg per 106 cells), and Goat Anti-Mouse FITC-IgG (Servicebio, Wuhan, China, #SF131; 1:200 dilution). All antibodies were diluted according to the manual from the manufacturer’s website. Dead cells and doublets were removed by dead-cell dye staining (Zombie Aqua Fixable Viability Kit, BioLegend, CA, USA, #B297827).

### Isolation of primary mouse hepatocytes and hepatic macrophages

Six- to eight-week-old male mice were infused with D-Hanks’ balanced solution via the portal vein for 3–5 min. After the color of the liver changed to beige or light brown, 0.05% IV collagenase (Sigma, Cat#C4-BIOC) was perfused into the liver. After cracks appeared on the surface of the liver, perfusion was stopped immediately, and the liver was excised in ice-cold DMEM containing 10% FBS. Cells from digested livers were isolated, suspended in DMEM, filtered through a 100-μm cell strainer, and centrifuged at 50 × *g* for 5 min at 4 °C. The pellet, representing the hepatocytes, was washed with DMEM twice and cultured in William’s Medium E (Thermo Fisher Scientific, Cat# 12551032) containing 10% FBS on collagen-coated plates. The supernatant, which was enriched in nonparenchymal cells, was loaded on a double-layer discontinuous iodixanol gradient of 11.5% and 20% OptiPrep (Sigma, D1556) and centrifuged at 1400 × *g* for 17 min at 4 °C without applying the centrifuge brake. The upper layer of 20% OptiPrep was collected and allowed to attach onto cell culture plates in DMEM with 10% FBS. Kupffer cells (KCs) and monocyte-derived macrophages (MDMs) were purified by FACS (KCs: F4/80^hi^CD11b^lo^CLEC4F^+^Ly6c^−^; MDMs: F4/80^lo^CD11b^hi^CLEC4F^−^Ly6c^+^) and cultured in 1640 medium.

### Coimmunoprecipitation (Co-IP)

Cells were lysed in Co-IP buffer (pH 7.5, 1% Triton X-100, 150 mM NaCl, protease inhibitor cocktail, 20 mM Tris, and 1 mM EDTA) on ice for 20 min. Cell supernatant (200 μl) was collected and then incubated with control IgG, primary antibodies against Flag (4 μg, BioLegend, USA, # 637303) or PirB (4 μg, Thermo Fisher Scientific, MA, USA, Cat#MA5-24049) with gentle rocking at 4 °C overnight followed by 1-h incubation with Protein A/G (MedChem Express, NJ, USA, #HY-K0202) at 4 °C. After three washes with co-IP lysis buffer, protein samples were collected BCby boiling in 3× SDS loading buffer and subjected to western blotting.

### Glutathione S-transferase (GST) pulldown assay

Purified Flag-tagged proteins were prepared by Cusabio Technology, Inc.(Wuhan, China) (details listed in supplementary method). GST pull-down assays were carried out using the Pierce GST Protein Interaction Pull-Down Kit (Thermo Fisher Scientific) according to the manufacturer’s instructions. Eluted protein was determined by immunoblotting using anti-GST (Cell Signaling Technology, China, Cat# 2625, 1:500 dilution), anti-His (Abcam, UK, #ab18184, 1:1,000 dilution), and anti-Flag (BioLegend, USA, # 637303, 1:500 dilution).

### Coculture of hepatocytes and monocyte-derived macrophages (MDMs)

Primary hepatocytes (5 × 10^5^) were seeded in the lower chamber of 24-well Transwell plates (Corning, NY, USA, #3450) 24 h before 2 × 10^5^ primary mouse MDMs were seeded in the upper chamber. Half an hour later, the hepatocytes and MDMs were incubated with or without 250 μM palmitate (PA) (Kunchuang Biotechnology, Xian, China, #KCKJ008) and/or 40 nM recombinant ANGPTL8 (rANGPTL8) (Cusabio Biotechnology Co., Ltd., Wuhan, China) at 37 °C for 18 h. For neutralizing antibody experiments, anti-TNFα (Thermo Fisher Scientific, MA, USA, #AMC3012), anti-IL-1β (Thermo Fisher Scientific, MA, USA, #16-7012-81) and anti-IL-6 (Thermo Fisher Scientific, MA, USA, #16-7061-81) were present at 1 μg/ml, and anti-PirB was present at 10 μg/ml. After incubation, media and cells were collected for analysis of triglyceride (TG) accumulation and inflammatory cytokine production. The mRNA expression of cytokines in the hepatocytes (the lower plates) and MDMs (the upper wells) was detected.

### Plasmid transfection

Plasmids encoding mouse full-length ANGPTL8, truncated ANGPTL8, and PirB ectodomain were synthesized by Obio Technology, Inc. (Shanghai, China). Briefly, the sequences encoding full-length ANGPTL8 (16–198) or its truncation (16–55, 55–198, and 130–198) were cloned into the pcDNA3.1-flag vector and the sequences encoding PirB ectodomain (22–643) were cloned into the pcDNA3.1-myc vector. All transient transfections were conducted using Lipofectamine 3000 (Invitrogen, CA, USA) according to the manufacturer’s instructions. After 24 h transfection, the medium was removed, and cells were lysed in 1 ml of ice-cold lysis buffer (pH 7.5, 1% Triton X-100, 150 mM NaCl, protease inhibitor cocktail, 20 mM Tris, and 1 mM EDTA).

### Lentivirus transduction

Lentiviral particles were prepared by Shanghai Genechem Co., Ltd. (Shanghai, China). The shRNA sequences targeting *PirB* were as follows: (shRNA#1, 5′-gcCCAGTGTATATGCTACTCT-3′; shRNA#2, 5′-acAGAATATGAACAAGCAGAA-3′; shRNA#3, 5′-ccTTCTGTCATGTCAAGGGAA-3′). The shRNA sequences targeting *PirA* were as follows: (shRNA#1, 5′-CAGGAGGACTAAGGTGACTTT-3′; shRNA#2, 5′-CAGGACCACAGAAGTTCTCAT-3′; shRNA#3, 5′-GCCGAGCTATGACAGGTTCAT-3′). The shscramble sequence was 5′-TTCTCCGAACGTGTCACGT-3′. The mouse macrophage cell line RAW264.7 was purchased from Cell Bank of the Chinese Academy of Sciences (catalog number: SCSP-5036). RAW264.7 were transinfected with the lentiviral particles for 12 h, and medium containing lentiviruses was then removed and changed to RPMI 1640 medium (HyClone, UT, USA, Cat# SH30027.01) with 10% FBS (Gibco, NY, USA, Cat# 10099-141C). After 72 h of culture, cells were selected using puromycin (8 μg/ml, Solarbio, Beijing, China, Cat# P8230) for 1 week and then maintained with 4 μg/ml puromycin.

### Antibody blockade assay

MDMs were pretreated with control IgG (10 µg/ml), anti-integrin-α5β1 antibody (10 µg/ml, Millipore, CA, USA, Cat# MAB2514), and anti-PirB ectodomain antibody (10 µg/ml, Thermo Fisher Scientific, MA, USA, Cat# MA5-24049) for 30 min before incubation with 20 nM rANGPTL8 for 24 h to detect cytokine expression and with 10 nM rANGPTL8 for 6 h to detect migration. hMDMs were pretreated with control IgG (10 µg/ml) and anti-LILRB2 antibody (5 µg/ml, R&D Systems, MN, USA, Cat#MAB2078) for 30 min before incubation with 20 nM rANGPTL8 for 24 h to detect cytokine expression and with 5 nM rANGPTL8 for 6 h to detect migration.

### Signaling pathway inhibitors

MDMs (mouse) and hMDMs were pretreated with inhibitors of ERK1/2 (U0126; 10 nM, MedChem Express, NJ, USA, Cat# HY-12031), P38 (SB203580; 10 nM, MedChem Express, NJ, USA, Cat# HY-10256), AKT (MK-2206; 10 nM, MedChem Express, NJ, USA, Cat# HY-10358) or P65 (Bay11-7082; 10 nM, MedChem Express, NJ, USA, Cat# HY-13453) for 30 min before incubation with 20 nM rANGPTL8 for 24 h to detect cytokine expression and with 10 nM (MDMs) and 5 nM (hMDMs) rANGPTL8 for 6 h to detect migration.

### RNA isolation and real-time quantitative PCR (RT–qPCR)

Total RNA was extracted using TRIzol (Thermo Fisher Scientific, MA, USA, Cat# 15596018) following the manufacturer’s instructions and quantified through use of a Nanodrop. To prepare RNA for PCR analysis, 1 μg of total RNA was converted to cDNA using Hifair® II 1st Strand cDNA Synthesis SuperMix (Yeasen, Shanghai, China, Cat#11120ES60). RT–qPCR was performed using the qPCR Hieff UNICON® qPCR SYBR Green Master Mix (Low Rox) (Yeasen, Shanghai, China, Cat#11199ES08).Relative mRNA levels were calculated using the 2^−ΔΔCt^ method and normalized to GAPDH mRNA levels. The gene-specific primer sequences are listed in Supplementary Table 1.

### Western blotting

Cells were washed with PBS three times and collected with RIPA lysis buffer (Beyotime, Shanghai, China, Cat# P0013B) according to the manufacturer’s protocol. Equal amounts of protein (20 μg) were loaded into each lane, separated through 10% SDS polyacrylamide gel electrophoresis, and electrotransferred to PVDF membranes. The PVDF membranes were blocked in blocking buffer for 2 h at RT, followed by incubation with the following antibodies overnight at 4 °C: mouse anti-β-Actin (Proteintech, Wuhan, China, Cat# 66009-1-Ig, 1:1000 dilution); rabbit anti-ANGPTL8 (Abcam, UK, Cat# ab180915, 1:1000 dilution), anti-ERK1/2 (Cell Signaling Technology, MA, USA, Cat# 4695T, 1:1000 dilution), anti-phospho-ERK1/2 (Thr202/Tyr204; Cell Signaling Technology, MA, USA, Cat# 4370T, 1:1000 dilution), anti-p38-MAPK (Cell Signaling Technology, MA, USA, Cat# 8690T, 1:1000 dilution), and anti-phospho-p38-MAPK (Thr180/Tyr182; Cell Signaling Technology, MA, USA, Cat# 4511T, 1:1000 dilution), anti-AKT (Cell Signaling Technology, MA, USA, Cat# 4691S, 1:1000 dilution), anti-phospho-AKT (Ser473; Cell Signaling Technology, MA, USA, Cat# 4060T, 1:1000 dilution), anti-NF-κB-p65 (Cell Signaling Technology, MA, USA, Cat# 8242T, 1:1000 dilution), anti-phospho- NF-κB-p65 (Ser536; Cell Signaling Technology, MA, USA, Cat# 3033T, 1:1000 dilution); mouse monoclonal anti-(His)6 antibody (Abcam, UK, Cat#ab237339, 1:2000 dilution); anti-SHP1 (Cell Signaling Technology, Shanghai, China, Cat# 3759, 1:1,000 dilution), anti-SHP2 (Cell Signaling Technology, Shanghai, China, Cat# 3397, 1:1000 dilution) and anti-phospho-tyrosine (Cell Signaling Technology, Shanghai, China, Cat# 9411 1:2000 dilution); anti-His (Abcam, UK, Cat# ab18184, 1:1000 dilution); anti-Flag (BioLegend, USA, # 637303, 1:500 dilution); and rat anti-PirB (Thermo Fisher Scientific, MA, USA, Cat# MA5-24049, 1:500 dilution). The blots were subsequently rinsed with TBST three times and incubated with peroxide-conjugated secondary antibodies for 90 min. The secondary antibodies used in this study were Peroxidase AffiniPure goat anti-rabbit-IgG (H + L) (Cell Signaling Technology, Shanghai, China, Cat#7074), goat anti-rat-IgG (H + L) (Cell Signaling Technology, Shanghai, China, Cat#7077), and goat anti-mouse-IgG (H + L) (Cell Signaling Technology, Shanghai, China, Cat#7076). Secondary antibodies were used at a 1:3000 dilution. The protein bands were visualized and detected using an enhanced chemiluminescence system.

### Cell Oil Red O staining

Cells were washed twice with PBS and fixed with 4% paraformaldehyde for 30 min. After two washes in PBS, cells were stained for 30 min in a freshly diluted Oil Red O solution (Sigma, MO, USA, Cat#O1391). The dishes were then rinsed in PBS and observed with a light microscope.

### Liver histology assessment and Oil Red O staining

For hematoxylin-eosin (H&E) staining and Sirius red staining, liver samples were harvested, paraformaldehyde-fixed and paraffin-embedded. For each mouse, three tissue sections from different hepatic lobes were prepared for H&E staining and Sirius red staining. Sliced sections with a thickness of 4 μm were stained with hematoxylin and eosin and then observed under a light microscope. For Oil Red O staining, frozen sections were stained with Oil Red O for 10 min, washed, counterstained with hematoxylin for 20 s, and then observed under a light microscope.

### Biochemical assays

Liver and serum triglyceride (TG) levels were assayed using a TG assay kit (Nanjing Jiancheng Bioengineering Institute, Nanjing, China, Cat# A110-1-1). Liver and serum triglyceride (TC) levels were assayed using a TC assay kit (Nanjing Jiancheng Bioengineering Institute, Nanjing, China, Cat# A111-1-1). Plasma LDL, VLDL, HDL, and NEFA levels were assayed using assay kits (Nanjing Jiancheng Bioengineering Institute, Nanjing, China, Cat# A113-1-1; H249-1-1; A112-1-1; and A042-2-1). Liver function was evaluated by measuring the serum levels of alanine aminotransferase (ALT) and aspartate aminotransferase (AST) using commercial kits (Nanjing Jiancheng Bioengineering Institute, Nanjing, China, Cat# C009-2-1 for ALT and Cat# C010-2-1 for AST). All assays and data analyses were performed according to the manufacturer’s protocol.

### Enzyme-linked immunosorbent assay (ELISA)

Commercially available mouse ELISA kits were used to measure ANGPTL8 (EIAab Science Inc., Wuhan, China, Cat# E11644m), interleukin (IL)-6 (Elabscience Biotechnology Co., Ltd., Wuhan, China, Cat# E-EL-M0044c), tumor necrosis factor (TNF)-α (Elabscience Biotechnology Co., Ltd., Wuhan, China, Cat# E-EL-M0049c), IL-1β (Elabscience Biotechnology Co., Ltd., Wuhan, China, Cat# E-EL-M0037c), and insulin (Elabscience Biotechnology Co., Ltd., Wuhan, China, Cat# CEA448Mu) levels in the serum or cell supernatants according to the manufacturer’s instructions.

### Liver immunofluorescence

The paraffin-embedded sections were deparaffinized and stained with primary antibodies against ANGPTL8 Rabbit pAb (Thermo Fisher Scientific, USA, Cat#PA5-38043, 1:100 dilution) and PirB Rat mAb (Thermo Fisher Scientific, USA, Cat#MA5-24049, 1:50 dilution) overnight at 4 °C. After washing in phosphate-buffered saline (PBS), sections were visualized by Alexa Fluor-conjugated secondary antibody (Servicebio, Wuhan, China, Cat# GB25303, 1:200 dilution) or Cy3-conjugated secondary antibody (Servicebio, Wuhan, China, Cat# GB21303, 1:200 dilution). Nuclear counterstaining was performed using 4’,6-diamidino-2-phenylindole (DAPI).

### Liver immunohistochemistry

The paraffin-embedded sections were deparaffinized and rehydrated. The sections were incubated with primary antibodies against F4/80 (Cell Signaling Technology, MA, USA, Cat# 70076T; 1:100 dilution), and CD11b (Servicebio, Wuhan, China, Cat# GB11058; 1:100 dilution) for 2 h at room temperature and washed three times with PBS. Then, the sections were incubated with horseradish peroxidase-conjugated secondary antibodies (Servicebio, Wuhan, China, Cat# GB23303, 1:200 dilution) for 1 h at room temperature, followed by 3,3’-diaminobenzidine (DAB) development, hematoxylin staining, dehydration, and mounting.

### Terminal deoxyribonucleotide transferase (TdT)-mediated dUTP nick end labeling (TUNEL) assay

In situ detection of DNA fragments by TUNEL was performed using a one-step TUNEL apoptosis assay kit (Beyotime, Shanghai, China, Cat#C1089) according to the manufacturer’s instructions.

### Immunocytochemistry

Cells were seeded in a 24-well plate with coverslips. For colocalization analysis, MDMs and KCs were treated with exogenous rANGPTL8 (20 nM) or Scrambled ANGPTL8 for 30 min. For nuclear translocation of NF-κB subunit p65, MDMs were treated with rANGPTL8 (40 nM) for 2 h. After treatment, the cells on the coverslips were fixed with 4% paraformaldehyde at room temperature for 10 min and then permeabilized with 0.1% Triton X-100 for 15 min. A blocking step was performed with 5% bovine serum albumin (BSA) for 1 h. Subsequently, the cells were incubated with the primary antibodies overnight at 4 °C. After washing, the cells were incubated with appropriate fluorescent secondary antibodies for 1 h. After three washes, the cells on the coverslips were incubated with DAPI (5 μg/mL) at room temperature for 5 min and observed under a fluorescence microscope. Antibodies used in this study included PE-conjugated anti-Flag mAb (BioLegend, USA, Cat# 637310, 1:100 dilution), anti-PirB Rat mAb (Thermo Fisher Scientific, USA, Cat#MA5-24049, 1:100 dilution), anti-LILRB2 Mouse mAb (R&D Systems, MN, USA, Cat#MAB2078, 1:50 dilution), Alexa Fluor-conjugated secondary antibody (Servicebio, Wuhan, China, Cat# GB25301 and GB22302, 1:500 dilution).

### Recombinant ANGPTL8 protein treatment

Before rANGPTL8 treatment, cells were serum-starved for 12 h. Then, the mouse MDMs were stimulated with 5 nM, 20 nM, and 40 nM rANGPTL8 protein (Cusabio,Wuhan, China) or scrambled ANGPTL8 for an additional 24-h period for detecting cytokines and 6-h, 12-h, 24-h period for migration assays. Human MDMs were stimulated with 5 nM rANGPTL8 protein or scrambled ANGPTL8 for an additional 24-h period for detecting cytokines and 6-h or 12-h period for migration assays.

### Transwell migration assay

Primary mouse MDMs (5 × 10^5^) were seeded with 100 μl of serum-free Dulbecco’s modified Eagle medium (DMEM) atop 8-µm polycarbonate filter inserts in Transwell chamber plates (Corning, NY, USA, #3422), and 500 μl of serum-free DMEM was added to the lower chamber. After incubation for 30 min, PBS or 5, 20, or 40 nM rANGPTL8 protein was added to the lower chamber. After incubation for 6, 12 or 24 h, cells that had not migrated were removed with a cotton swab from the upper surface of filters, and cells that had migrated to the lower surface of the membrane were stained with crystal violet at 25 °C for 10 min, washed with PBS, and subsequently observed with a light microscope.

### PirB ectodomain protein injection

The recombinant protein for PirB (sPirB) was prepared by Cusabio (Wuhan, China, # CSB-MP012941MO2). The PirB ectodomain DNA fragment was synthesized by CUSABIO (Wuhan, China). The DNA fragment was inserted into the pSecTag2A plasmid, which contained a (His)_6_ tag. The plasmid was transformed into HEK293 cells, and the recombinant protein was purified using a Ni^2+^-nitrilotriacetic acid superflow agarose column. CDHFD-fed *loxp* and *Angptl8*^*HepKO*^ mice received an intravenous injection of 1 mg/kg sPirB once every three days for three months.

### Recombinant proteins

Full-length amino acid sequence and detailed information were listed at Supplementary information.

### RNA-sequencing analysis

The mRNA of the MDMs was extracted and sent to Shanghai Genminix Informatics Co., Ltd. for mRNA sequencing. Differential gene expression analysis was completed using the limma R package (version 3.44.3). The fold change (FC) in expression of each gene was log2 transformed and further analyzed using RStudio version 1.1.442 (RStudio, Inc., USA).

### Homology comparison

Protein sequence comparisons were performed by Megalign and Jalview. Molecular docking was performed by AutoDock.

### Human peripheral blood and liver samples

Heparinized blood samples obtained from the Tongji Hospital of Wuhan were collected from NAFLD patients (from the endocrinology department) and healthy volunteers (from the physical examination center). Primary liver specimens were obtained from hemangioma surgical resection. All samples were used for cell extraction and consent to publish was obtained. Sample sex is selected randomly. Two-step collagenase perfusion technique was used to isolate human liver cells. The initial perfusion was performed for at least 20 min or until the whole blood has been washed out. Collagenase perfusion was carried out for 10–20 min. Then, ongoing digestion by the remaining collagenase is prevented by a stop solution [100 ml fetal calf serum (FCS) in 500 ml sterile PBS] added to the cell suspension released from the digested tissue. We isolated human peripheral blood mononuclear cells (PBMCs) from the blood using Ficoll density gradient centrifugation. Monocytes were purified from PBMCs using a monocyte isolation kit (Solarbio, Beijing, China, P5290) according to the manufacturer’s instructions. The cells were cultured with 0.1 μg/ml PMA (Sigma, MedChem Express, NJ, USA, Cat#HY-18739) to generate monocyte-derived macrophages (hMDMs). The human study was approved by the Ethical Committee of Tongji Hospital, Tongji Medical College, Huazhong University of Science and Technology (IRB ID: TJ-C20220801) and was conducted according to the principles of the Declaration of Helsinki. Written informed consent was obtained from the patients.

### Statistical analyses

For animal studies, mice were randomized by body weight prior to dietary challenge, and no blinding was performed for subsequent analyses. Statistical analyses were performed with Prism 7 (GraphPad Software, Inc., USA) or RStudio version 1.1.442 (RStudio, Inc., USA). For data with a normal distribution and homogeneity of variance, one-way ANOVA was performed for comparisons among more than two groups. Two-tailed Student’s t tests were performed to evaluate significant differences between two groups. The statistical tests used for each experiment are indicated in the figure legends. All data are expressed as the mean ± s.e.m. unless otherwise indicated. Differences for which *P* < 0.05 were considered significant. All in vitro experiments were performed in triplicate. Animal feeding, treatments and histological analyses were performed in a single-blinded fashion.

### Reporting summary

Further information on research design is available in the Nature Portfolio Reporting Summary linked to this article.

## Supplementary information

Supplementary Information

Reporting Summary

## Data Availability

All methods and data supporting the findings are available within the manuscript or supplementary information. The RNA-Seq data generated in this study have been deposited in the National Center for Biotechnology Information Sequence Read Archive database under accession code PRJNA992231. The RNA-Seq data shown in Fig. 1a–d were published^13,14^, and the primary data are available in the National Center for Biotechnology Information Gene Expression Omnibus database with accession code GSE126848, GSE167523, GSE130970 and GSE136103. Source data are provided with this paper in Source Data file. Uncropped blots are available in Source Data file. Source data are provided with this paper.

## Source data

Source Data
