# Supplementary material for: LILRB2/PirB mediates macrophage recruitment in fibrogenesis of nonalcoholic steatohepatitis
Source: Nat Commun. 2023 Jul 22;14:4436. doi: 10.1038/s41467-023-40183-3 (PMC10363120; doi:10.1038/s41467-023-40183-3)
Supplement: Supplementary file 1 — Supplementary Information [file 41467_2023_40183_MOESM1_ESM.pdf]

Title

LILRB2/PirB mediates macrophage recruitment in fibrogenesis of nonalcoholic steatohepatitis

Authors
Dan-Pei Li[1,2 #], Li Huang[1,2 #], Ran-Ran Kan[1,2], Xiao-Yu Meng[1,2], Shu-Yun Wang[1,2], Hua-Jie Zou[1,2], Ya-Ming Guo[1,2], Pei-Qiong Luo[1,2], Li-Meng Pan[1,2], Yu-Xi Xiang[1,2], Bei-Bei Mao[1,2], Yu-Yu Xie[1,2], Zhi-Han Wang[1,2], Min Yang[1,2], Rui He[1,2], Yan-Yang[1,2], Zhe-Long Liu[1,2], Jun-Hui Xie[1,2], De-Lin Ma[1,2], Ben-Ping Zhang[1,2], Shi-Ying Shao[1,2], Xi Chen[1,2], Si-Miao Xu[1,2], Wen-Tao He[1,2], Wen-Jun Li[3], Yong Chen[1,2*] & Xue-Feng, Yu[1,2*]

[1]Division of Endocrinology, Department of Internal Medicine, Tongji Hospital, Tongji Medical College, Huazhong University of Science and Technology, Wuhan, China
[2]Branch of National Clinical Research Center for Metabolic Diseases, Hubei, China
[3]Computer Center, Tongji Hospital, Tongji Medical College, Huazhong University of Science and Technology, Wuhan, China

# These authors contributed equally to this study.
* Corresponding authors.

Supplementary file includes:

Supplementary Figures 1 to 8

Supplementary information

Supplementary Table 1

Supplementary Figures

Supplementary Fig. 1 ANGPTL1-8 expression in the liver of human and murine NASH.

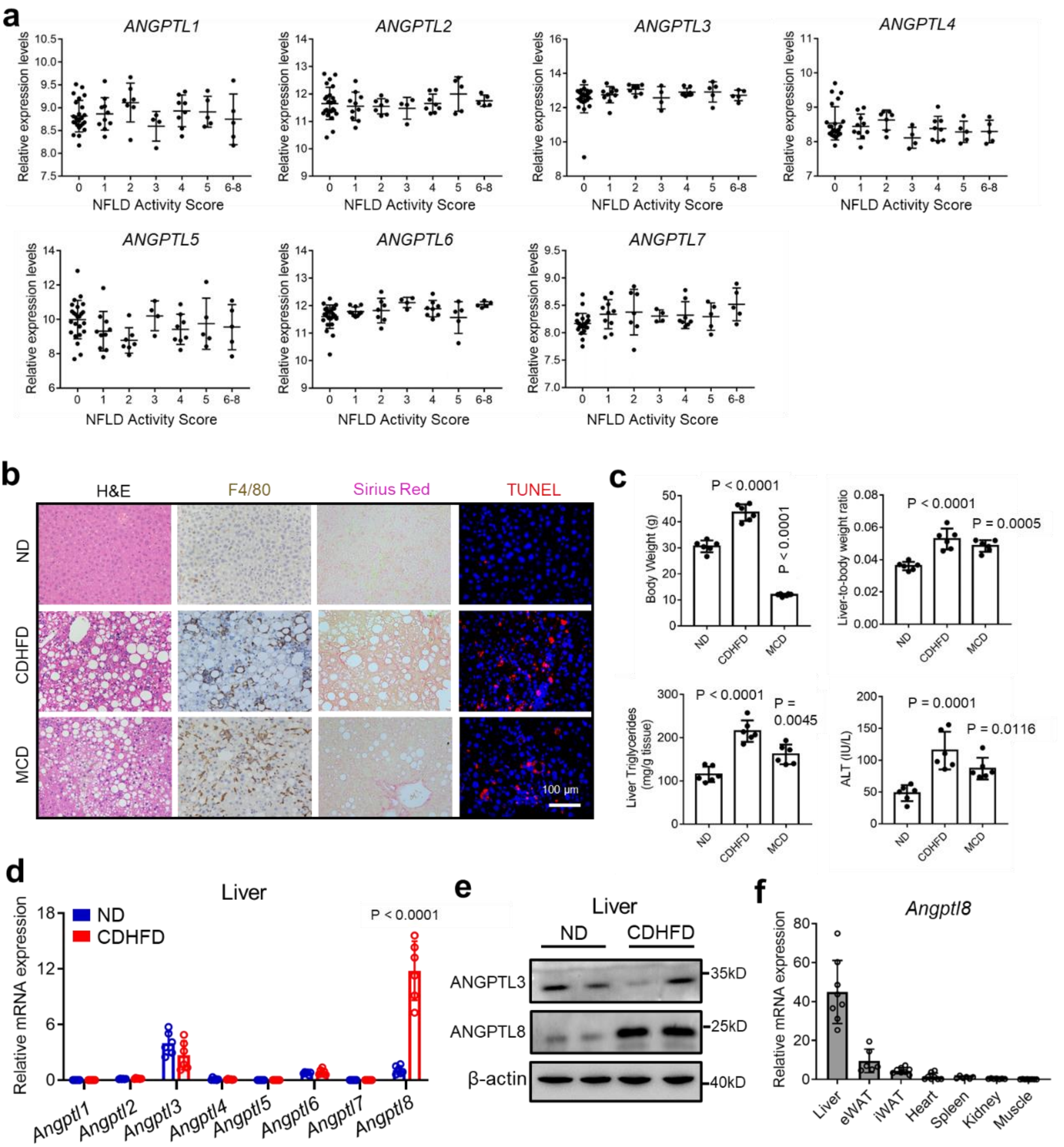

(a) ANGPTL1-7 mRNA levels in the livers of NASH patients (n = 63 biologically independent samples) from GEO datasets (GSE136103). (b) NASH characterization by H&E, F4/80, Sirius Red, and TUNEL staining of the indicated groups. Scale bar, 100 μm. (c) Body weight, liver-to-body weight ratio, ALT, and liver TG content of the indicated groups (n = 6 mice/group). (d) ANGPTL1-8 mRNA levels in the livers of the indicated mouse groups (n = 6 mice/group). (e) Representative western blot of liver ANGPTL3 and ANGPTL8 protein of the indicated groups. (f) ANGPTL8 expression distribution in mice (n = 6 or 8 mice). All samples are biologically independent replicates and n indicates the number of biologically independent samples examined. Data shown are representative of three independent experiments with similar results (b, e). The data are shown as the mean ± s.e.m. and were statistically analyzed by one-way ANOVA with Tukey's multiple-comparison

test (a and c); All the p values were two-sided and adjustments were made for multiple comparisons. kD, relative molecular weight in kilodalton; ND: normal diet, CDHFD: choline-deficient high-fat diet, and MCD, methionine-choline deficient. Source data are available as a Source Data file.

Supplementary Fig. 2 Hepatic PirB is mainly expressed in monocyte-derived macrophages (MDMs).

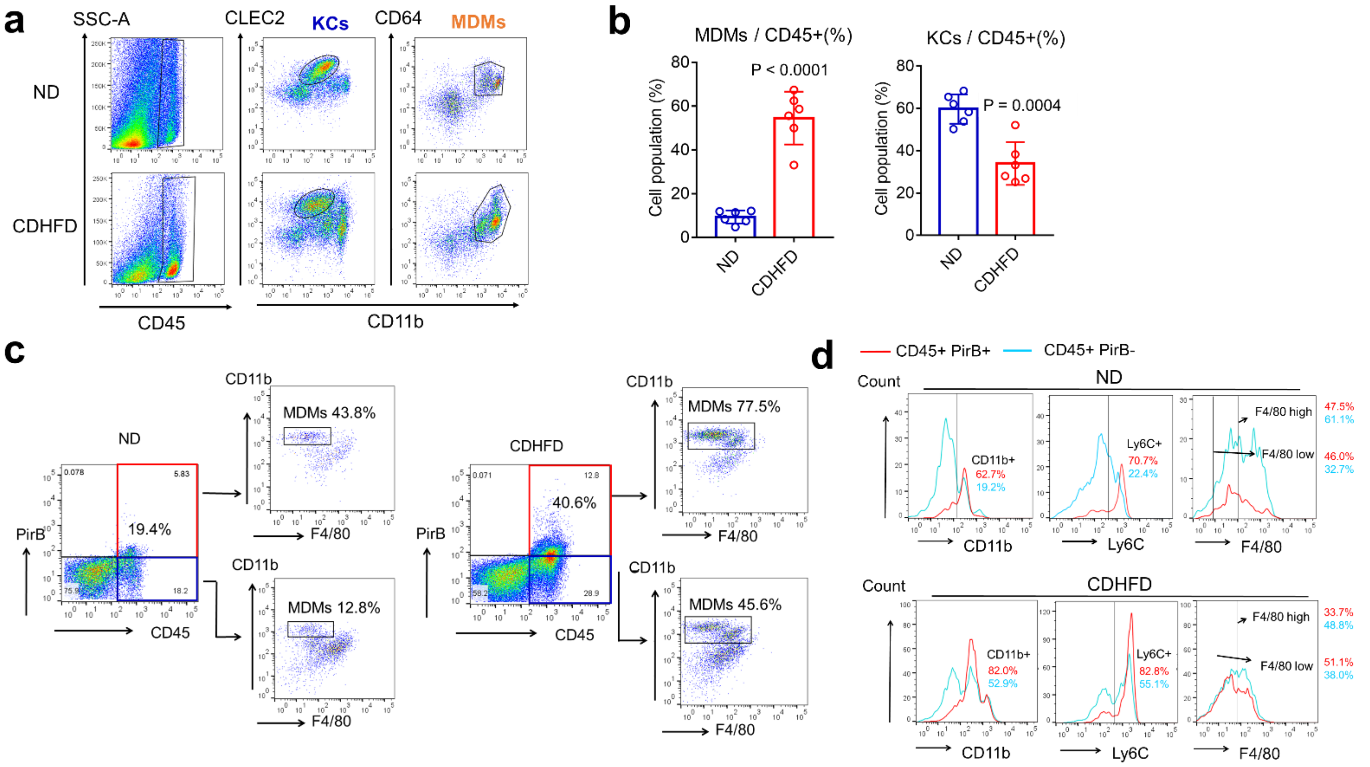

(a) The gating strategy for flow cytometry analyses. To discriminate KCs from MDMs, we first gated on KCs defined as CD11b^{lo} CLEC2^{hi} cells. Among the remaining cells, we identified CLEC2^{lo} CD11b^{+} CD64^{+} cells as MDMs. (b) FACS quantification of hepatic macrophages (n = 6 biologically independent samples). The data are shown as the mean ± s.e.m. and were statistically analyzed by two-tailed Student's t test. All the p values were two-sided (c) We merged PirB+ CD45+ (red) and PirB- CD45+ (blue) cells into a whole group. In the PirB+ CD45+ fraction, MDMs accounted for 43.8% (ND, left) and 77.5% (CDHFD, right). The ratio of PirB+ MDMs/PirB- MDMs increased from 1.1 to 1.5 during NASH. (d) The proportions of CD11b^{+}, Ly6C^{+}, and F4/80^{lo} cells were greater in the PirB+ CD45+ fraction than in the PirB- CD45+ fraction. ND, normal diet; CDHFD, choline-deficient high-fat diet; MDMs, monocyte-derived macrophages; KCs, Kupffer cells. Source data are available as a Source Data file.

Supplementary Fig. 3 ANGPTL8 binds to PirB on MDMs via its C-terminal domain.

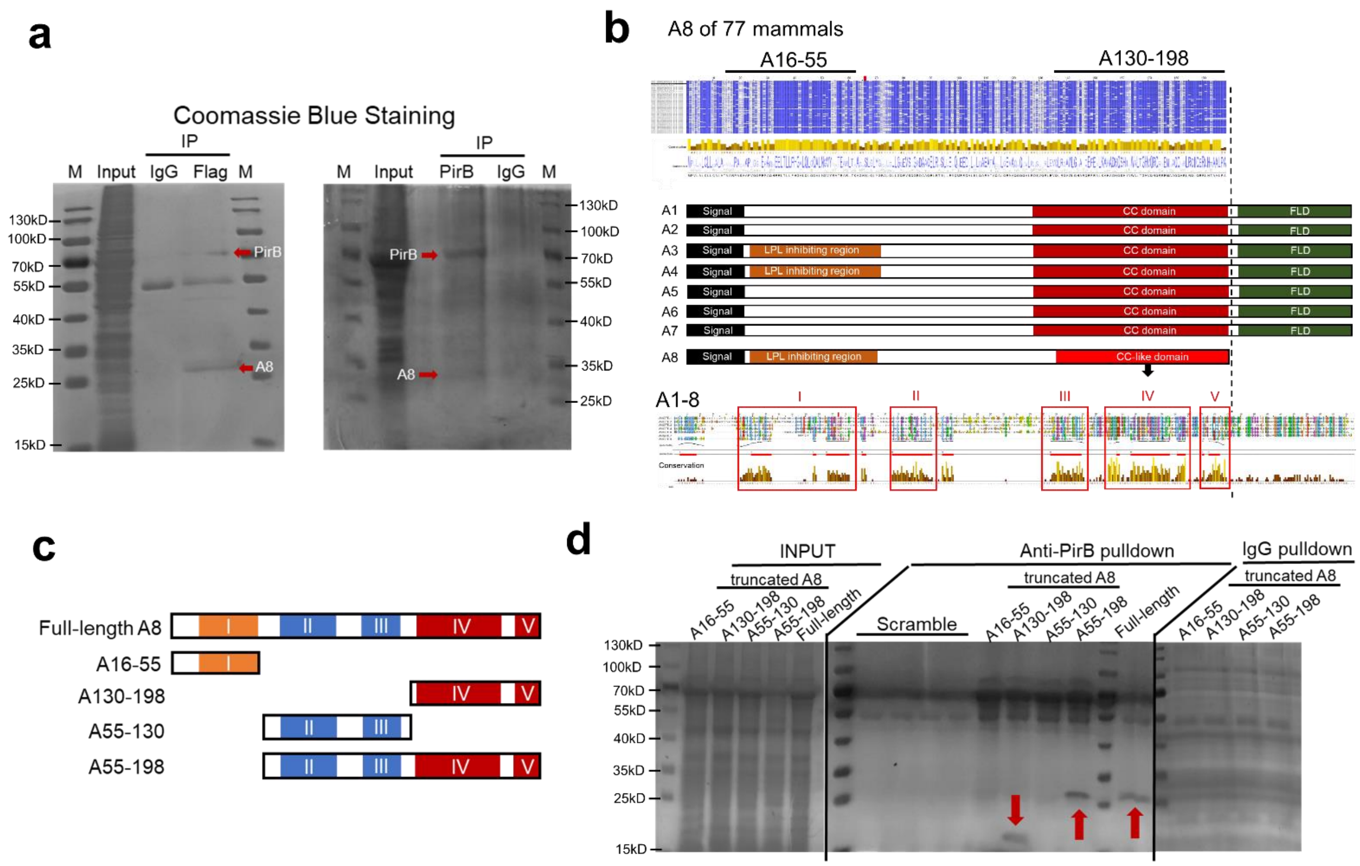

(a) Coomassie blue-stained gel of the indicated protein, which was coimmunoprecipitated using anti-Flag (for ANGPTL8-flag, right) and anti-pirB (left) antibodies and identified via mass spectrometry (MS). IgG was used as a control; M, marker. (b, upper) Comparison of the amino acid sequences of ANGPTL8 in 77 mammalian species reveals high identity in 16-55 and 130-198. A, amino acid; the shades of blue indicate the degree of similarity, where dark blue indicates high similarity. (b, lower) Homology of the ANGPTL family members identifies the CC-like domain of ANGPTL8 in the A130-198 conserved region. Conservation is indicated by rectangular bars. A1-8, ANGPTL1-8; Black: signal peptide; Orange: region mediating lipoprotein lipase (LPL) binding and inhibiting its activity; Dark red: coiled-coil (CC) domain; Red: CC-like domain; Green: fibronectin-like domain (FLD). (c) Schematic showing full-length and truncated ANGPTL8. (d) Coomassie blue-stained gel of immunoprecipitation by PirB. RAW264.7 cells stably expressing truncated proteins were established by transfection with an overexpression plasmid. Red arrows show the indicated pulldown fraction; IgG was used as a control. Data shown are representative of three independent experiments with similar results (a, d). kD, relative molecular weight in kilodalton.

Supplementary Fig. 4 ANGPTL8 promotes MDM migration

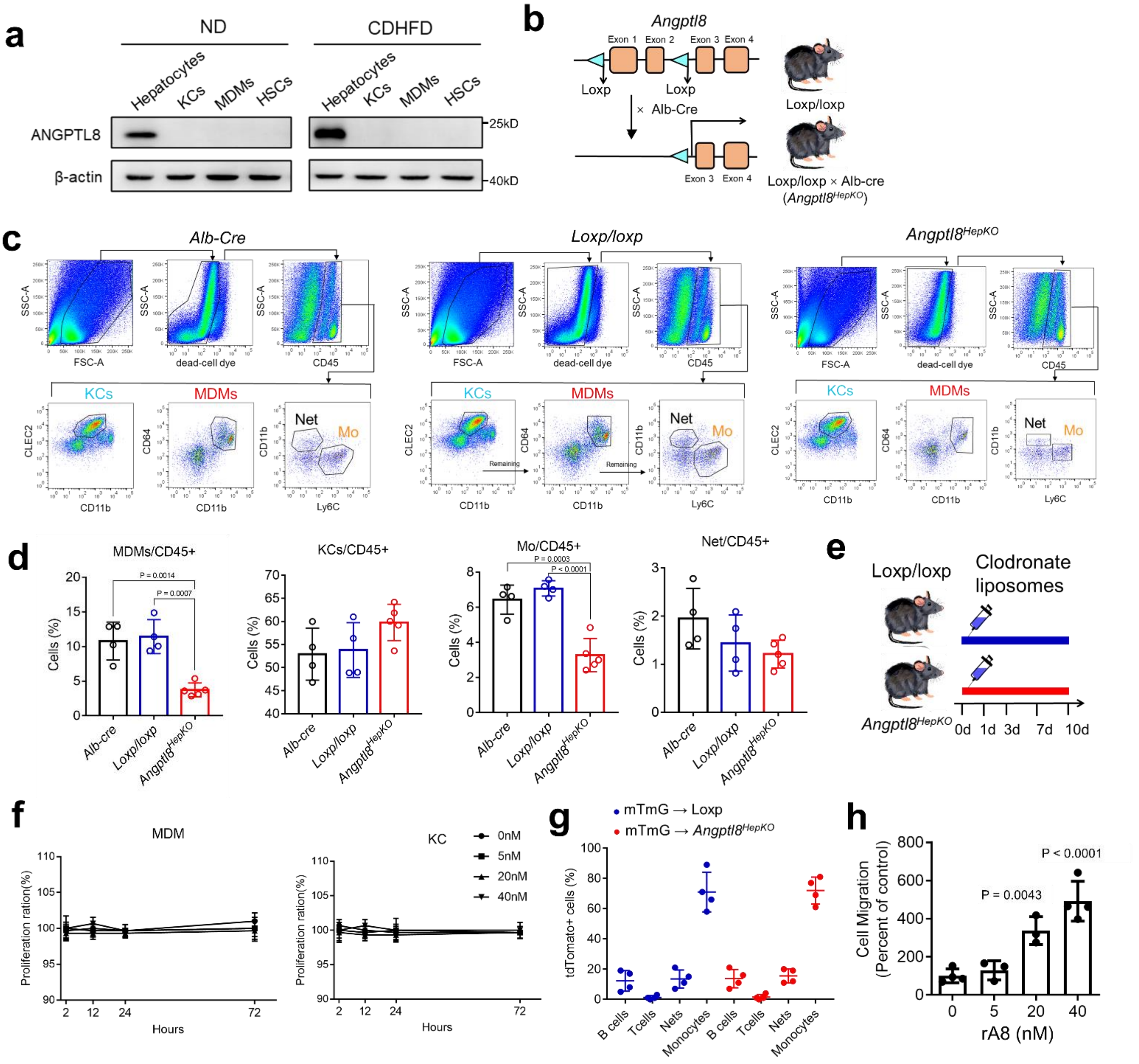

(a) Representative western blot for intracellular ANGPTL8 protein expression in hepatic cells. (b) The strategy used to generate *Angptl8*^{HepKO} mice. (c) The gating strategy for flow cytometry analyses. To discriminate KCs from MDMs, we first gated on KCs defined as CD11b^{lo} CLEC2^{hi} cells. Among the remaining cells, we identified CLEC2^{lo} CD11b+ CD64+ cells as MDMs. Finally, Ly6C+ CD11b^{int} monocytes and Ly6C^{int} CD11b^{hi} neutrophils were identified in the CLEC2- CD64- fraction. (d) FACS quantification of hepatic macrophages (n = 4 biologically independent samples). (e) Experimental scheme to deplete hepatic macrophages. (f) Cell proliferation of KCs and MDMs after recombinant ANGPTL8 protein (rANGPTL8) treatment (n = 4 independent experiments). The numbers of viable cells were measured using a CCK-8 kit. (g) Blood total leukocytes in chimerism, T cells, B cells, neutrophils (Nets), and monocytes 2 weeks after bone marrow transplantation (BMT) (n = 4 biologically independent samples). (h) Quantification of MDM migration to different concentrations of rANGPTL8 over time (n = 3 or 4 independent experiments). The data are shown as the mean ± s.e.m. and were statistically analyzed by one-way ANOVA with Tukey's multiple-comparison test; All the p values were two-sided and adjustments were made for multiple comparisons. Data shown are representative of three independent experiments with similar results (a, c). kD, relative molecular weight in kilodalton. Source data are available as a Source Data file.

Supplementary Fig. 5 ANGPTL8-induced MDM activation promotes lipid accumulation in hepatocytes.

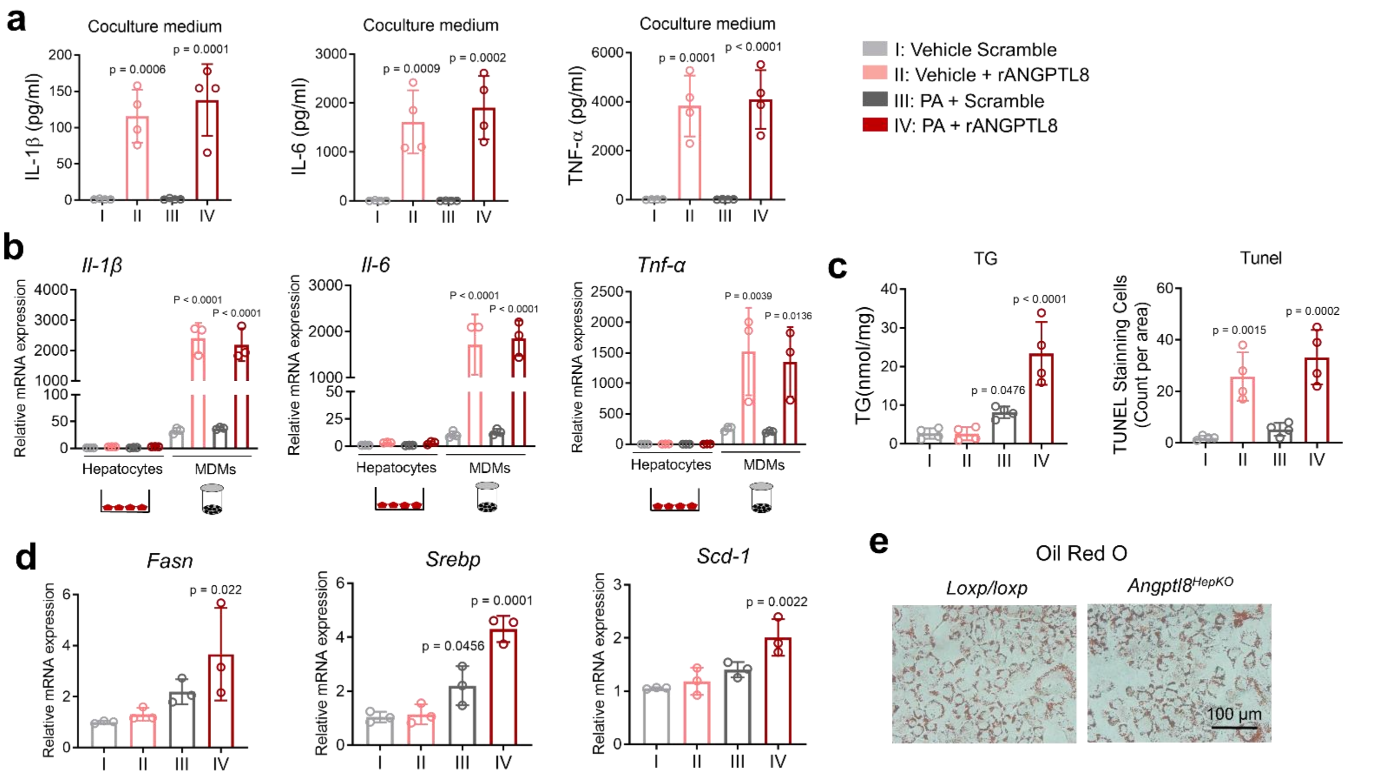

Hepatocytes were cultured in the presence or absence of MDMs and treated with or without rANGPTL8 and PA, after which cytokine levels (a, n = 4 independent experiments) from the coculture media and their corresponding mRNA expression (b, n = 3 independent experiments) of hepatocytes and MDMs were tested; TG content, TUNEL staining (c, n = 4 independent experiments), and lipogenesis genes of hepatocytes were measured (d, n = 3 independent experiments). (e) Oil Red O staining of primary hepatocytes from *loxp/loxp* and *Angptl8*^{HepKO} mice. Scale bar, 100 μm. The data are shown as the mean ± s.e.m. and were statistically analyzed by one-way ANOVA with Tukey's multiple-comparison test; All the p values were two-sided and adjustments were made for multiple comparisons. PA, palmitate; KCs, Kupffer cells; MDMs, monocyte-derived macrophages. Data shown are representative of three independent experiments with similar results (e). Source data are available as a Source Data file.

Supplementary Fig. 6 PirB and its downstream signalling molecules mediate ANGPTL8-induced cytokine production and the migration of MDMs.

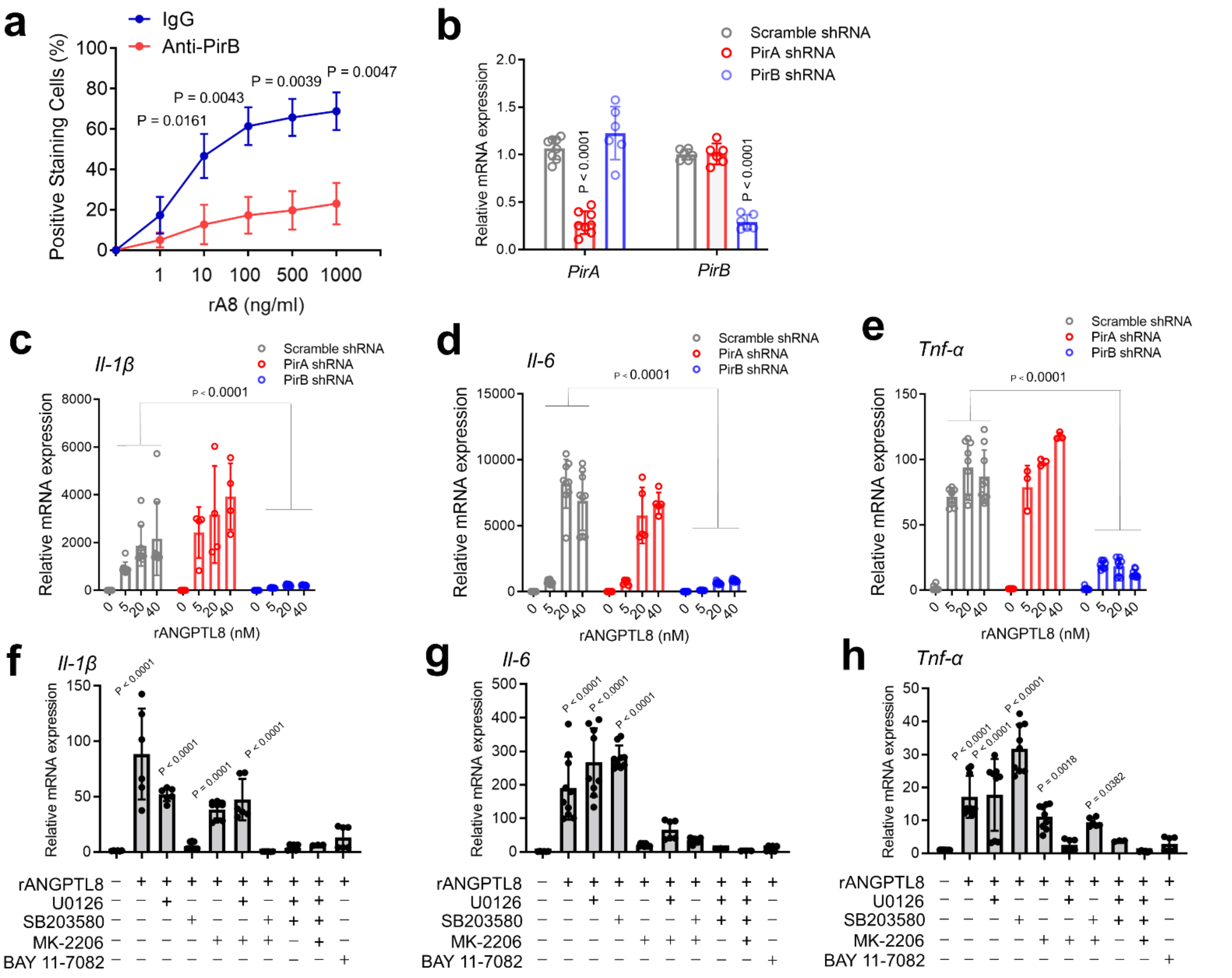

(a) Quantification of flag-tagged (red) rA8 on MDMs pretreated with 1 µg/ml indicated antibodies at different rA8 concentrations (n = 3 independent experiments). IgG was used as a control. (b-e) mRNA expression of PirA/PirB (b) and cytokines (c-e) in RAW264.7 cells transfected with lentiviruses containing shRNA (n = 9 cells examined over 3 independent experiments) of scramble, PirA (n = 3 or 5 cells examined over 3 independent experiments) and PirB (n = 9 cells examined over 3 independent experiments). (f-h) Cytokine expression in MDMs pretreated with phosphorylation inhibitors before ANGPTL8 treatment (n = 6 or 9 cells examined over 3 independent experiments). The data are shown as the mean ± s.e.m. and were statistically analyzed by one-way ANOVA with Tukey's multiple-comparison test; All the p values were two-sided and adjustments were made for multiple comparisons. MDMs, monocyte-derived macrophages; rANGPTL8 (rA8), recombinant ANGPTL8 protein. Source data are available as a Source Data file.

Supplementary Fig. 7 Hepatocyte-specific ANGPTL8 knockout reduces MDM infiltration into the liver during NASH.

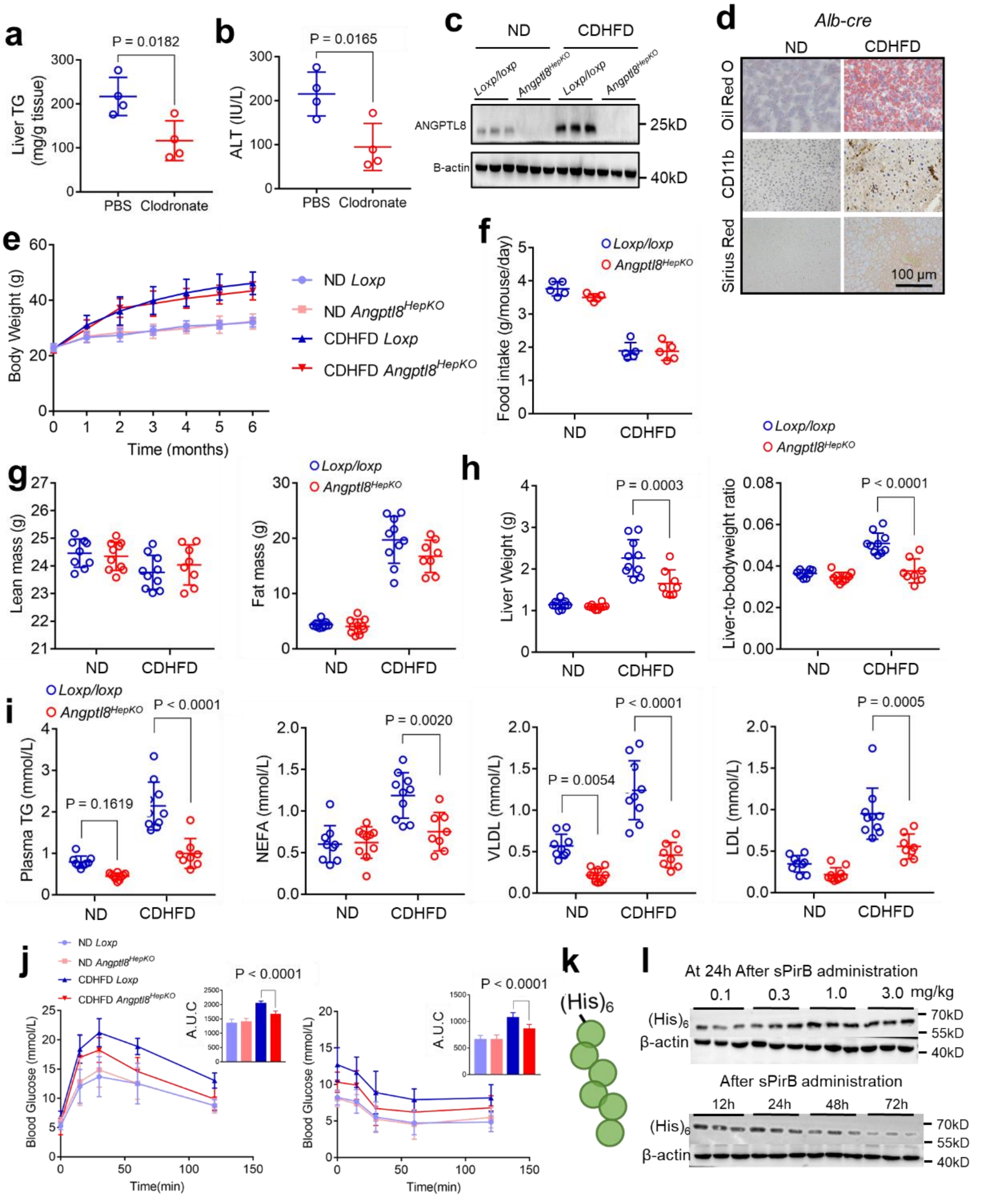

(a) Liver TG contents and (b) ALT of NASH mice with macrophage depletion (n = 4 mice/group). (c) Western blot showing ANGPTL8 protein expression in the livers of ND/CDHFD-fed loxp and *Angptl8^{HepKO}* mice. (d) Representative liver images of liver sections. Scale bar, 100 μm. (e-j) Body weight (e), food intake (f), body composition (g), liver weight and liver-to-body weight ratio (h), plasma lipid profile (i), IPGTT and ITT (j) of the indicated groups (n = 8 or 10 mice/group). (k) Structure of recombinant soluble PirB ectodomain (sPirB) protein. (l) Western blot of $(His)_6$-flagged protein in livers from mice injected with sPirB. The data are shown

as the mean ± s.e.m. and were statistically analyzed by two-tailed Student's t-test (a and b) or one-way ANOVA with Tukey's multiple-comparison test (d-i); All the p values were two-sided and adjustments were made for multiple comparisons. Data shown are representative of three independent experiments with similar results (c, d, l). kD, relative molecular weight in kilodalton; sPirB, recombinant soluble PirB ectodomain protein; ND: normal diet; CDHFD: choline-deficient high-fat diet. Source data are available as a Source Data file.

Supplementary Fig. 8 ANGPTL8 mediates human peripheral blood monocyte via LILRB2

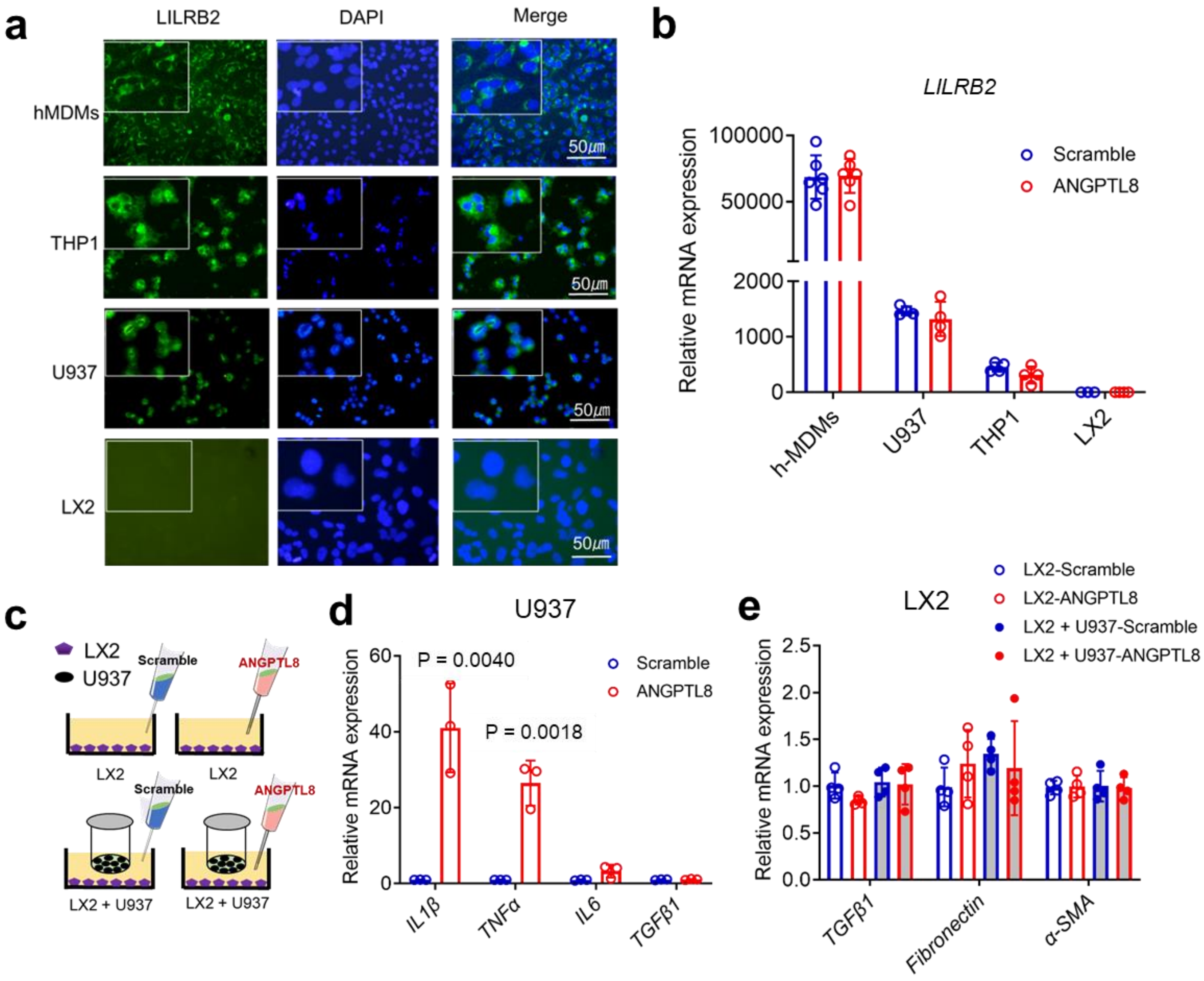

(a) Immunocytochemistry staining showing LILRB2 expression on indicated cells. Scale bar, 50 µm. (b) Relative mRNA expression of LILRB2 in the indicated cells (n = 6 for MDMs; 4 for U943 and THP1; 3 for LX2; n indicates the number of independent experiments). (c) Experimental scheme to coculture hepatocytes with hMDMs. (d) mRNA expression of proinflammatory cytokines in human U937-derived macrophages (n = 3 independent experiments). (e) mRNA expression of fibrogenic genes in LX2 cells (n = 4 independent experiments). The data are shown as the mean ± s.e.m. and were statistically analyzed by two-tailed Student's t test (b, d) or one-way ANOVA with Tukey's multiple-comparison test (e); All the p values were two-sided and adjustments were made for multiple comparisons. Data shown are representative of three independent experiments with similar results (a). hMDMs, human monocyte-derived macrophages. Source data are available as a Source Data file.

Supplemental information

Recombinant proteins

All the recombinant proteins were tested by SDS–PAGE quantitative densitometry by Coomassie blue staining, and the purities were 95%-98%. No aggregated or sticky protein was found. Proteins were reconstituted in deionized sterile water to a concentration of 0.1-1.0 mg/mL, and were added 5% glycerol (final concentration) and aliquoting for long-term storage at -20°C/-80°C.

Recombinant mouse ANGPTL8 protein

(1)

MRFPSIFTAVLFAASSALAAPVNTTTEDETAQIPAEAVIGYSDLEGDFDVAVLPFSNSTNNGLLFINTTIASIAAKEEGVSLEKR (signal peptide cleavage site) EAEAYVHHHHHHEFRT+VRPAPVAPLGGPEPAQYEELTLLFHGALQLGQALNGVYRATEARLTEAGHSLGLYDRALEFLGTEVRQGQDATQELRTSLSEIQVEEDALHLRAEATARSLGEVARAQQALRDTVRRLQVQLRGAWLGQAHQEFETLKARADKQSHLLWALTGHVQRQQREMAEQQQWLRQIQQRLHTAALPA+DYKDDDDK

α-factor secretion signal: MRFPSIFTAVLFAASSALAAPVNTTTEDETAQIPAEAVIGYSDLEGDFDVAVLPFSNSTNNGLLFINTTIASIAAKEEGVSLEKR (signal peptide cleavage site) EAEA

6xHis: HHHHHH

FLAG-tag: DYKDDDDK

Linker: RT

(2)

MRFPSIFTAVLFAASSALAAPVNTTTEDETAQIPAEAVIGYSDLEGDFDVAVLPFSNSTNNGLLFINTTIASIAAKEEGVSLEKR (signal peptide cleavage site) EAEAYVHHHHHHEFRT+VRPAPVAPLGGPEPAQYEELTLLFHGALQLGQALNGVYRATEARLTEAGHSLGLYDRALEFLGTEVRQGQDATQELRTSLSEIQVEEDALHLRAEATARSLGEVARAQQALRDTVRRLQVQLRGAWLGQAHQEFETLKARADKQSHLLWALTGHVQRQQREMAEQQQWLRQIQQRLHTAALPA

α-factor secretion signal: MRFPSIFTAVLFAASSALAAPVNTTTEDETAQIPAEAVIGYSDLEGDFDVAVLPFSNSTNNGLLFINTTIASIAAKEEGVSLEKR (signal peptide cleavage site) EAEA

6xHis: HHHHHH

Linker: RT

Mutant ANGPTL8 protein

(1) Mut1:

MRFPSIFTAVLFAASSALAAPVNTTTEDETAQIPAEAVIGYSDLEGDFDVAVLPFSNSTNNGLLFINTTIASIAAKEEGVSLEKR (signal peptide cleavage site) EAEAYVHHHHHHEFRT+VRPAPVAPLGGPEPAQYEELTLLFAGALALGAALNGVYRAAEARLTEAGHSLGLYDRALEFLGTEVRQGQDATQELRTSLSEIQVEEDALHLRAEATARSLGEVARAQQALRDTVRRLQVQLRGAWLGQAHQEFETLKARADKQSHLLWALTGHVQRQQREMAEQQQWLRQIQQRLHTAALPA

α-factor secretion signal: MRFPSIFTAVLFAASSALAAPVNTTTEDETAQIPAEAVIGYSDLEGDFDVAVLPFSNSTNNGLLFINTTIASIAAKEEGVSLEKR (signal peptide cleavage site) EAEA

6xHis: HHHHHH

Linker: RT

(2) Mut2:

MRFPSIFTAVLFAASSALAAPVNTTTEDETAQIPAEAVIGYSDLEGDFDVAVLPFSNSTNNGLLFINTTIASIAAKEEGVSLEKR (signal peptide cleavage site) EAEAYVHHHHHHEFRT+VRPAPVAPLGGPEPAQYEELTLLFHGALQLGQALNGVYRATEARLTEAGHSLGLYDRALEF

LGTEVRQGQAATQALATSLSEIAVEEDALHLRAEATARSLGEVARAQQALRDTVRRLQVQLRGAWLGQAHQEFETLKA
RADKQSHLLWALTGHVQRQQREMAEQQQWLRQIQQRLHTAALPA
α-factor secretion signal:
MRFPSIFTAVLFAASSALAAPVNTTTEDETAQIPAEAVIGYSDLEGDFDVAVLPFSNSTNNGLLFINTTIASIAAKEEGVSLEK
R (signal peptide cleavage site) EAEA
6xHis: HHHHHH
Linker: RT
(3) Mut3:
MRFPSIFTAVLFAASSALAAPVNTTTEDETAQIPAEAVIGYSDLEGDFDVAVLPFSNSTNNGLLFINTTIASIAAKEEGVSLEK
R (signal peptide cleavage site)
EAEAYVHHHHHHEFRT+VRPAPVAPLGGPEPAQYEELTLLFHGALQLGQALNGVYRATEARLTEAGHSLGLYDRALEFL
GTEVRQGQDATQELRTSLSEIQVEEDALHLRAEATARALGEVAAAAQALRDAVRRLQVQLRGAWLGQAHQEFETLKA
RADKQSHLLWALTGHVQRQQREMAEQQQWLRQIQQRLHTAALPA
α-factor secretion signal:
MRFPSIFTAVLFAASSALAAPVNTTTEDETAQIPAEAVIGYSDLEGDFDVAVLPFSNSTNNGLLFINTTIASIAAKEEGVSLEK
R (signal peptide cleavage site) EAEA
6xHis: HHHHHH
Linker: RT
(4) Mut4:
MRFPSIFTAVLFAASSALAAPVNTTTEDETAQIPAEAVIGYSDLEGDFDVAVLPFSNSTNNGLLFINTTIASIAAKEEGVSLEK
R (signal peptide cleavage site)
EAEAYVHHHHHHEFRT+VRPAPVAPLGGPEPAQYEELTLLFHGALQLGQALNGVYRATEARLTEAGHSLGLYDRALEFL
GTEVRQGQDATQELRTSLSEIQVEEDALHLRAEATARSLGEVARAQQALRDTVRRLQVQLRGAALGQAAQEFATLKAA
ADAQSHLLWALTGHVQRQQREMAEQQQWLRQIQQRLHTAALPA
α-factor secretion signal:
MRFPSIFTAVLFAASSALAAPVNTTTEDETAQIPAEAVIGYSDLEGDFDVAVLPFSNSTNNGLLFINTTIASIAAKEEGVSLEK
R (signal peptide cleavage site) EAEA
6xHis: HHHHHH
Linker: RT
(5) Mut5:
MRFPSIFTAVLFAASSALAAPVNTTTEDETAQIPAEAVIGYSDLEGDFDVAVLPFSNSTNNGLLFINTTIASIAAKEEGVSLEK
R (signal peptide cleavage site)
EAEAYVHHHHHHEFRT+VRPAPVAPLGGPEPAQYEELTLLFHGALQLGQALNGVYRATEARLTEAGHSLGLYDRALEFL
GTEVRQGQDATQELRTSLSEIQVEEDALHLRAEATARSLGEVARAQQALRDTVRRLQVQLRGAWLGQAHQEFETLKAR
ADKQSHLLWALTGHVQRQAREMAEQAQWLRQAAQRLHTAALPA
α-factor secretion signal:
MRFPSIFTAVLFAASSALAAPVNTTTEDETAQIPAEAVIGYSDLEGDFDVAVLPFSNSTNNGLLFINTTIASIAAKEEGVSLEK
R (signal peptide cleavage site) EAEA
6xHis: HHHHHH
Linker: RT

Scrambled ANGPTL8 protein

(1)
MRFPSIFTAVLFAASSALAAPVNTTTEDETAQIPAEAVIGYSDLEGDFDVAVLPFSNSTNNGLLFINTTIASIAAKEEGVSLEK
R (signal peptide cleavage site)
EAEAYVHHHHHHEFRT+SAEERYHSLARKTHGYGELWVRKDALLVGGVFLTVRRQQATTSRAGHLERQVGEQPLALPA
RLIALTLQSATLLQRQLAGARYERQFRAQELAAPLQLDLLAADDEQQRLAEGRQQGQRRQEPDHAHMGQQELTVAGGT
LQTLLVELLVEAHEPASHTIAEFLPLATWWAREVAQGQQRNAQE+DYKDDDDK
α-factor secretion signal:

MRFPSIFTAVLFAASSALAAPVNTTTEDETAQIPAEAVIGYSDLEGDFDVAVLPFSNSTNNGLLFINTTIASIAAKEEGVSLEK
R (signal peptide cleavage site) EAEA
6xHis: HHHHHH
FLAG-tag: DYKDDDDK
Linker: RT
(2)
MRFPSIFTAVLFAASSALAAPVNTTTEDETAQIPAEAVIGYSDLEGDFDVAVLPFSNSTNNGLLFINTTIASIAAKEEGVSLEK
R (signal peptide cleavage site)
EAEAYVHHHHHHEFRT+SAEERYHSLARKTHGYGELWVRKDALLVGGVFLTVRRQQATTSRAGHLERQVGEQPLALPA
RLIALTLQSATLLQRQLAGARYERQFRAQELAAPLQLDLLAADDEQQRLAEGRQQGQRRQEPDHAHMGQQELTVAGGT
LQTLLVELLVEAHEPASHTIAEFLPLATWWAREVAQGQQRNAQEα-factor secretion signal:
MRFPSIFTAVLFAASSALAAPVNTTTEDETAQIPAEAVIGYSDLEGDFDVAVLPFSNSTNNGLLFINTTIASIAAKEEGVSLEK
R (signal peptide cleavage site) EAEA
6xHis: HHHHHH
Linker: RT

Recombinant ectodomain protein of mouse PirB

METDTLLLWVLLLWVPGSTG (signal peptide cleavage site) DAAQPARRAVRSL+
SLPKPILRVQPDSVVSRRTKVTFLCEETIGANEYRLYKDGKLYKTVTKNKQKPENKAEFSFSNVDLSNAGQYRCSYSTQY
KSSGYSDLLELVVTGHYWTPSLLAQASPVVTSGGYVTLQCESWHNDHKFILTVEGPQKLSWTQDSQYNYSTRKYHALFS
VGPVTPNQRWICRCYSYDRNRPYVWSPPSESVELLVSGNLQKPTIKAEPGSVITSKRAMTIWCQGNLDAEVYFLHNEKSQ
KTQSTQTLQEPGNKGKFFIPSVTLQHAGQYRCYCYGSAGWSQPSDTLELVVTGIYEYYEPRLSVLPSPVVTAGGNMTLH
CASDFPYDKFILTKEDKKFGNSLDTEHISSSGQYRALFIIGPTTPTHTGAFRCYGYYKNAPQLWSVPSALQQILISGLSKKPS
LLTHQGHILDPGMTLTLQCFSDINYDRFALHKVGGADIMQHSSQQTDTGFSVANFTLGYVSSSTGGQYRCYGAHNLSSE
WSASSEPLDILITGQLPLTPSLSVQPNHTVHSGETVSLLCWSMDSVDTFILSKEGSAQQPLRLKSKSHDQQSQAEFSMSAV
TSHLSGTYRCYGAQDSSFYLLSSASAPVELTVSGPIETSTPPPTMSMPLGGLHMYLK+HHHHHH
Igκ leader: METDTLLLWVLLLWVPGSTG (signal peptide cleavage site) D
6xHis: HHHHHH

GST-PirB

MGSSHHHHHHSSGLVPRGSHMASMTGGQQMGRGSEFELMSPILGYWKIKGLVQPTRLLLEYLEEKYEEHLYERDEGDK
WRNKKFELGLEFPNLPYYIDGDVKLTQSMAIIRYIADKHNMLGGCPKERAEISMLEGAVLDIRYGVSRIAYSKDFETLKVD
FLSKLPEMLKMFEDRLCHKTYLNGDHVTHPDFMLYDALDVVLYMDPMCLDAFPKLVCFKKRIEAIPQIDKYLKSSKYIA
WPLQGWQATFGGGDHPPKSDLEVLFQGPLGSPEFRT+SLPKPILRVQPDSVVSRRTKVTFLCEETIGANEYRLYKDGKLYK
TVTKNKQKPENKAEFSFSNVDLSNAGQYRCSYSTQYKSSGYSDLLELVVTGHYWTPSLLAQASPVVTSGGYVTLQCES
WHNDHKFILTVEGPQKLSWTQDSQYNYSTRKYHALFSVGPVTPNQRWICRCYSYDRNRPYVWSPPSESVELLVSGNLQK
PTIKAEPGSVITSKRAMTIWCQGNLDAEVYFLHNEKSQKTQSTQTLQEPGNKGKFFIPSVTLQHAGQYRCYCYGSAGWS
QPSDTLELVVTGIYEYYEPRLSVLPSPVVTAGGNMTLHCASDFPYDKFILTKEDKKFGNSLDTEHISSSGQYRALFIIGPTTP
THTGAFRCYGYYKNAPQLWSVPSALQQILISGLSKKPSLLTHQGHILDPGMTLTLQCFSDINYDRFALHKVGGADIMQHS
SQQTDTGFSVANFTLGYVSSSTGGQYRCYGAHNLSSEWSASSEPLDILITGQLPLTPSLSVQPNHTVHSGETVSLLCWSMD
SVDTFILSKEGSAQQPLRLKSKSHDQQSQAEFSMSAVTSHLSGTYRCYGAQDSSFYLLSSASAPVELTVSGPIETSTPPPTM
SMPLGGLHMYLK
6xHis: HHHHHH
Thrombin site: LVPRGS
T7-tag: MASMTGGQQMG
GST-Tag:
MSPILGYWKIKGLVQPTRLLLEYLEEKYEEHLYERDEGDKWRNKKFELGLEFPNLPYYIDGDVKLTQSMAIIRYIADKHN
MLGGCPKERAEISMLEGAVLDIRYGVSRIAYSKDFETLKVDFLSKLPEMLKMFEDRLCHKTYLNGDHVTHPDFMLYDAL
DVVLYMDPMCLDAFPKLVCFKKRIEAIPQIDKYLKSSKYIAWPLQGWQATFGGGDHPPK
PSP (Prescission Protease): LEVLFQGP

Linker: RT

GST-Control

MGSSHHHHHHSSGLVPRGSHMASMTGGQQMGRGSEFELMSPILGYWKIKGLVQPTRLLLEYLEEKYEEHLYERDEGDK
WRNKKFELGLEFPNLPYYIDGDVKLTQSMAIIRYIADKHNMLGGCPKERAEISMLEGAVLDIRYGVSRIAYSKDFETLKVD
FLSKLPEMLKMFEDRLCHKTYLNGDHVTHPDFMLYDALDVVLYMDPMCLDAFPKLVCFKKRIEAIPQIDKYLKSSKYIA
WPLQGWQATFGGGDHPPKSDLEVLFQGPLGSPEFRT+TLLDGECLLRCITPREYAAQGWQHSGQKKMLTEDQAGNIDVE
NTSSEPTSTHMTLAGWSELRYLYDYLYTVVESIGLEITYSQSGQSQSHALTICESIGCGEPPFFIDPFKLLSTEHYPQLMRHI
YFGSFCQYPIVGVQYSVKLPYSCTKYGGGRPVSFPFGVSKYDWCASSDKKVQALSLSCNSSVIHAMAQPSLSDTAQRTPP
KIGTWNDQSLSPVVQHEDVMSKLVCRTSRGCLIQEYSPSHRLPTPYLDPNLLALFVSTDHPPRPLKSAGLSQKSQDLGDV
SPYNFGQKIYKLVLSEQTTRPKCPKPSDYTGTRLYTSFPTTQSVQNGGTNSKGLEYLLWSEVSSSGKESHQSSKSYSKLGT
TSAVGHSLCISLTTGVYNRLLTTTNTHIAQSSDRYQMEVYRENVGTAAMITTQNTPELEYDRLSKAEISDLQDSSSWKSVG
KLGGSSSIFGKSGSSRKAKFYTGVSASQNFNAVSQNTSLGYTKFQVYTPGLPQPDALHSWYLCAPNDYKVGCVHVYWT
NFGLETPYVWEFDELQAFLIAHTNVEILWQPTFPLYQDVISYPSGVMQPKSRQYFKVSGRSNHLVNLFTGLGHLMASHTI
KQPHASTPLLKAAA

6xHis: HHHHHH

Thrombin site: LVPRGS

T7-tag: MASMTGGQQMG

GST-Tag:

MSPILGYWKIKGLVQPTRLLLEYLEEKYEEHLYERDEGDKWRNKKFELGLEFPNLPYYIDGDVKLTQSMAIIRYIADKHN
MLGGCPKERAEISMLEGAVLDIRYGVSRIAYSKDFETLKVDFLSKLPEMLKMFEDRLCHKTYLNGDHVTHPDFMLYDAL
DVVLYMDPMCLDAFPKLVCFKKRIEAIPQIDKYLKSSKYIAWPLQGWQATFGGGDHPPK

PSP (Prescission Protease): LEVLFQGP

Linker: RT

Supplementary Table 1. Primer sequences

	Gene name	Primer sequences (5′-3′)
Mouse	*Angptl8*-F	CTCAATGGCGTGTACAGAGC
	Angptl8-R	TCGAAGGTGTAAAGCGTCCT
	Srebf-1-F	TCTGTGAGAAGGCCAGTGGGTA
	Srebf-1-R	GAGCTGTGGCCTCATGTAGGAATA
	Fasn-F	TGAATCAGCCCCACGCAGT
	Fasn-R	CCGAGTCAGTCTTGGAGGACAT
	Scd1-F	GTTAGCACCTTCTTGCGATACACT
	Scd1-R	GTGAAGTTGATGTGCCAGCG
	Il-1β-F	ATGGGCTGGACTGTTTCTAATG
	Il-1β-R	CTTGTGACCCTGAGCGACC
	Tnf-α-F	CCAGACCCTCACACTCAGATC
	Tnf-α-R	CACTTGGTGGTTTGCTACGAC
	Il-6-F	GTTCTCTGGGAAATCGTGGA
	Il-6-R	GGAAATTGGGGTAGGAAGGA
	PirB-F	TGCCACCCAGGAAGAAAGCCTA
	PirB-R	GCCCTCTTCTGCTTGTTCATATTC
	PirA1-F	CTGTGCCTCAGACTTTCACTACGA
	PirA1-R	AGCCCTGAGATGAGTATTTGTTGA
	Tgf-β1-F	CTCCCGTGGCTTCTAGTGC
	Tgf-β1-R	GCCTTAGTTTGGACAGGATCTG
	Gapdh-F	ACTCCCACTCTTCCACCTC
	Gapdh-R	TCTTGCTCAGTGTCCTTGC
	Angptl1-F	TCTGGAGCCCGAAAGTGACTAC
	Angptl1-R	AAATGCCATCTTGGTGCTTGC
	Angptl2-F	TCAACTTCTTCAGGAACTGGGAG
	Angptl2-R	AGTTGCCTTGGTTCGTCAGC
	Angptl3-F	AGATGACCTTCCTGCCGACTG
	Angptl3-R	CATGGACTGCCTGATTGGGTA
	Angptl4-F	GGATAGAGTCCCTGAAGGCCA
	Angptl4-R	TGAGCTGGGTCATCTTGGGA
	Angptl6-F	TGGCAGAGTGGAGTGTATGACC
	Angptl6-R	CAAAGCCCGCCTTGTAGTG
	Angptl7-F	GCCATCTATGACTGTTCTTCCCTG
	Angptl7-R	TGCTGCCAAACCCTTGCTTA
Human	*IL-1β*-F	CCTGTCCTGCGTGTTGAAAG
	IL-1β-R	GGGAACTGGGCAGACTCAAA
	IL-6-F	TTCGGTCCAGTTGCCTTCTC
	IL-6-R	GCCTCTTTGCTGCTTTCACA
	TNF-α-F	CCTCTCTCTAATCAGCCCTCTG
	TNF-α-R	GAGGACCTGGGAGTAGATGAG
	LILRB2-F	CACTGGACATCGACCCAGA
	LILRB2-R	GTCCATCTCCACCCCATCTT
	GAPDH-F	TCAAGAAGGTGGTGAAGCAGG
	GAPDH-R	TCAAAGGTGGAGGAGTGGGT
