# Supplementary material for: LILRB2/PirB mediates macrophage recruitment in fibrogenesis of nonalcoholic steatohepatitis
Source: Nat Commun. 2023 Jul 22;14:4436. doi: 10.1038/s41467-023-40183-3 (PMC10363120; doi:10.1038/s41467-023-40183-3)
Supplement: Supplementary file 2 — Reporting Summary [file 41467_2023_40183_MOESM2_ESM.pdf]

nature portfolio

Corresponding author(s): Yong Chen & Xue-Feng Yu

Last updated by author(s): Jul 09, 2023

Reporting Summary

Nature Portfolio wishes to improve the reproducibility of the work that we publish. This form provides structure for consistency and transparency in reporting. For further information on Nature Portfolio policies, see our Editorial Policies and the Editorial Policy Checklist.

Statistics

For all statistical analyses, confirm that the following items are present in the figure legend, table legend, main text, or Methods section.

n/a	Confirmed	
☐	☒	The exact sample size (*n*) for each experimental group/condition, given as a discrete number and unit of measurement
☐	☒	A statement on whether measurements were taken from distinct samples or whether the same sample was measured repeatedly
☐	☒	The statistical test(s) used AND whether they are one- or two-sided<br>*Only common tests should be described solely by name; describe more complex techniques in the Methods section.*
☒	☐	A description of all covariates tested
☒	☐	A description of any assumptions or corrections, such as tests of normality and adjustment for multiple comparisons
☐	☒	A full description of the statistical parameters including central tendency (e.g. means) or other basic estimates (e.g. regression coefficient) AND variation (e.g. standard deviation) or associated estimates of uncertainty (e.g. confidence intervals)
☐	☒	For null hypothesis testing, the test statistic (e.g. *F*, *t*, *r*) with confidence intervals, effect sizes, degrees of freedom and *P* value noted<br>*Give P values as exact values whenever suitable.*
☒	☐	For Bayesian analysis, information on the choice of priors and Markov chain Monte Carlo settings
☒	☐	For hierarchical and complex designs, identification of the appropriate level for tests and full reporting of outcomes
☒	☐	Estimates of effect sizes (e.g. Cohen's *d*, Pearson's *r*), indicating how they were calculated

Our web collection on statistics for biologists contains articles on many of the points above.

Software and code

Policy information about availability of computer code

Data collection	BD FACSAria III and BD FACSDiva 6.0 software was used for cell sorting. CytoFLEX (Beckman Coulter) and MACSQuantTM VYB were applied to acquire flow cytometry data. OLYMPUS Upright microscope BX53 was used to acquire microscopy images. Body composition were measured by Bruker SkyScan1276. Protein sequence comparisons were performed by DNAstar 7.0.1 (Megalign) and Jalview v2.10.5. Molecular docking was performed by AutoDock 4.2.6.
Data analysis	Statistical analyses were performed with Graphpad Prism 7.0 (GraphPad Software, Inc., USA) or RStudio version 1.1.442 (RStudio, Inc., USA). Immunoflurorescent images were processed and analysed using Image J1.46r. Flow cytometry analysis was performed using FlowJo v7.6.5.

For manuscripts utilizing custom algorithms or software that are central to the research but not yet described in published literature, software must be made available to editors and reviewers. We strongly encourage code deposition in a community repository (e.g. GitHub). See the Nature Portfolio guidelines for submitting code & software for further information.

Data

Policy information about availability of data

All manuscripts must include a data availability statement. This statement should provide the following information, where applicable:

- Accession codes, unique identifiers, or web links for publicly available datasets
- A description of any restrictions on data availability
- For clinical datasets or third party data, please ensure that the statement adheres to our policy

All methods and data supporting the findings are available within the manuscript or supplementary information. The RNA-Seq data generated in this study have been deposited in the National Center for Biotechnology Information Sequence Read Archive database under accession code PRJNA992231 (https://www.ncbi.nlm.nih.gov/sra). The RNA-Seq data used in this study are available in the National Center for Biotechnology Information Gene Expression Omnibus database under accession code GSE126848, GSE167523, GSE130970 and GSE136103 (https://www.ncbi.nlm.nih.gov/geo). Source data are provided with this paper. Uncropped blots are available in Supplementary Fig. 9.

Human research participants

Policy information about studies involving human research participants and Sex and Gender in Research.

Reporting on sex and gender	Sex: Man and weman; Sample sex is selected randomly.
Population characteristics	Age: 40-60 years old; Heparinized blood samples obtained from NAFLD patients and healthy volunteers. Primary liver specimens were obtained from haemangioma surgical resection.
Recruitment	Participants were recruited from the endocrinology department and the physical examination centre of the Tongji Hospital. Participants were recruited randomly and without self-selection
Ethics oversight	The Ethics Committee of the Tongji Hospital,Tongji Medical College, Huazhong University of Science and Technology (IRB ID: TJ-C20220801)

Note that full information on the approval of the study protocol must also be provided in the manuscript.

Field-specific reporting

Please select the one below that is the best fit for your research. If you are not sure, read the appropriate sections before making your selection.

☒ Life sciences ☐ Behavioural & social sciences ☐ Ecological, evolutionary & environmental sciences

For a reference copy of the document with all sections, see nature.com/documents/nr-reporting-summary-flat.pdf

Life sciences study design

All studies must disclose on these points even when the disclosure is negative.

Sample size	The sample size was chosen to assure significant statistical differences and reproducibility of the results. The maximum number of available mice for each experiment was used respecting the guidelines of animal welfare. For in vivo experiments, cohort size was determined by types of experiment and availability of animals as littermates were used in each experiment and our sample size ranging from 3-10 mice per group. For in vitro experiments. We repeated at least 3 times in each group. No statistical method was used to predetermine the sample size. The sample size was determined based on similar previous studies of our labratory and on previous experiments using similar methodologies. Detailed sample size were described in the figure legends.
Data exclusions	No data were excluded.
Replication	All data are the result of independently-repeated experiments with independent biological sample. Experiments were repeated independently at least twice with similar results in repeated experiments.
Randomization	Mice were randomized into the different groups.Cells and tissues from human donors were also randomized.
Blinding	Mice were randomized into the different groups. Cells and tissues from human donors were also randomized. The investigators were blinded to group allocation during data collection and analysis.

Reporting for specific materials, systems and methods

We require information from authors about some types of materials, experimental systems and methods used in many studies. Here, indicate whether each material, system or method listed is relevant to your study. If you are not sure if a list item applies to your research, read the appropriate section before selecting a response.

Materials & experimental systems

n/a	Involved in the study
☐	☒ Antibodies
☐	☒ Eukaryotic cell lines
☒	☐ Palaeontology and archaeology
☐	☒ Animals and other organisms
☒	☐ Clinical data
☒	☐ Dual use research of concern

Methods

n/a	Involved in the study
☒	☐ ChIP-seq
☐	☒ Flow cytometry
☒	☐ MRI-based neuroimaging

Antibodies

Antibodies used

Anti-p-ERK1/2 (Thr202/Tyr204) Rabbit mAb supplier: Cell Signaling Technology, catalog number: 4370T, clone number: D13.14.4E
Anti-ERK1/2 Rabbit mAb supplier: Cell Signaling Technology, catalog number: 4695T, clone number: 137F5
Anti-p-p38 MAPK(Thr180/Tyr182) Rabbit mAb supplier: Cell Signaling Technology, catalog number: 4511T, clone number: D3F9
Anti-p38 MAPK Rabbit mAb supplier: Cell Signaling Technology, catalog number: 8690T, clone number: D13E1
Anti-p-AKT (Ser473) Rabbit mAb supplier: Cell Signaling Technology, catalog number: 4060T, clone number: D9E
Anti-AKT Rabbit mAb supplier: Cell Signaling Technology, catalog number: 4691S, clone number: C67E7
Anti-p-NF-κB p65 (Ser536) Rabbit mAb supplier: Cell Signaling Technology, catalog number: 3033T, clone number: 93H1
Anti-NF-κB p65 Rabbit mAb supplier: Cell Signaling Technology, catalog number: 8242T, clone number: D14E12
Anti-ANGPTL8 Rabbit pAb supplier: Thermo Fisher Scientific, catalog number: PA5-38043
Anti-ANGPTL8 Rabbit pAb supplier: Abcam, catalog number: ab180915
Anti-PirB Rat mAb supplier: Thermo Fisher Scientific, catalog number: MA5-24049, clone number: 326414
Anti-SHP1 Rabbit mAb supplier: Cell Signaling Technology, catalog number: 3759, clone number: C14H6
Anti-SHP2 Rabbit mAb supplier: Cell Signaling Technology, catalog number: 3397, clone number: D50F2
Anti-phospho-tyrosine supplier: Cell Signaling Technology, catalog number: 9411, clone number: P-Tyr-100
Anti-LILRB2 Mouse mAb supplier: R&D Systems, catalog number: MAB2078, clone number: 287219
Anti-His Mouse mAb supplier: Abcam, catalog number: ab18184, clone number: HIS.H8
Anti-Flag Rat mAb supplier: BioLegend, catalog number: 637303, clone number: L5
Anti-GST Rabbit supplier: Cell Signaling Technology, catalog number: 2625, clone number: 91G1
Anti-TNFα Rabbit pAb supplier: Thermo Fisher Scientific, catalog number: AMC3012
Anti-IL-1β Armenian hamster mAb supplier: Thermo Fisher Scientific, catalog number: 16-7012-81, clone number: B122
Anti-IL-6 Rat mAb supplier: Thermo Fisher Scientific, catalog number: 16-7061-81, clone number: MP5-20F3
Anti-F4/80 Rabbit mAb supplier: Cell Signaling Technology, catalog number: 70076T, clone number: D2S9R
Anti-CD11c Rabbit pAb supplier: Servicebio, catalog number: GB11059,
Anti-CD68 Rabbit pAb supplier: Servicebio, catalog number: GB113109,
Anti-CD11b Rabbit pAb supplier: Servicebio, catalog number: GB11058,
PE anti-mouse F4/80 supplier: BioLegend, catalog number: 123109, clone number: BM8
FITC anti-mouse CD11c supplier: BioLegend, catalog number: 117305, clone number: N418
APC anti-mouse CD206 supplier: BioLegend, catalog number: 141707, clone number: C068C2
FITC anti-mouse CD45 supplier: BioLegend, catalog number: 103108, clone number: 30-F11
PE anti-mouse CLEC2 supplier: BioLegend, catalog number: 146104, clone number: 17D9/CLEC-2
BV421 anti-mouse F4/80 supplier: BioLegend, catalog number: 123127, clone number: BM8
Pe/Cy7 anti-mouse CD64 supplier: BioLegend, catalog number: 139313, clone number: X54-5/7.1
Percp/Cy5.5 anti-mouse/human CD11b supplier: BioLegend, catalog number: 101228, clone number: M1/70
APC/Cy7 anti-mouse PirB supplier: R&D, catalog number: FAB2754S, clone number: 326414
APC anti-mouse Ly6C supplier: BioLegend, catalog number: 128016, clone number: HK1.4
PE anti-human CD14 supplier: BD Biosciences Pharmingen, catalog number: 555398, clone number: M5E2
APC anti-mouse Ly6G/Ly6C supplier: BioLegend, catalog number: 108411, clone number: RB6-8C5
PerCP anti-mouse CD19 supplier: BioLegend, catalog number: 115531, clone number: 6D5
APC anti-mouse CD3 supplier: BioLegend, catalog number: 100235, clone number: 17A2
Anti-β-Actin Mouse mAb supplier: Proteintech, catalog number: 66009-1-Ig, clone number: 2D4H5
Anti-integrin-α5β1 Rat mAb supplier: Millipore, catalog number: MAB2514, clone number: BMB5
Alexa Fluor-conjugated secondary antibody supplier: Servicebio, catalog number: GB25303, GB25301, and GB22302
Cy3-conjugated secondary antibody supplier: Servicebio, catalog number: GB21303
Peroxidase AffiniPure goat anti-rabbit-IgG (H+L) supplier: Cell Signaling Technology, catalog number: 7074
Peroxidase AffiniPure goat anti-rat-IgG (H+L) supplier: Cell Signaling Technology, catalog number: 7077
Peroxidase AffiniPure goat anti-mouse-IgG (H+L) supplier: Cell Signaling Technology, catalog number: 7076

Validation

All antibodies used are commercially available and validated by the vendor for the assay and species used in this study. Specific validation information for each antibody is available on the vendors website. The specificity of each primary flow cytometry antibody was validated by staining directly against species-matched isotype antibodies and unstained controls. Validation of each primary antibody used for immunostaining was performed by comparison to species-matched isotype antibodies and unstained controls.
Anti-p-ERK1/2 (Thr202/Tyr204) Rabbit mAb https://www.cellsignal.cn/products/primary-antibodies/phospho-p44-42-mapk-erk1-2-thr202-tyr204-d13-14-4e-xp-rabbit-mab/4370
Anti-ERK1/2 Rabbit mAb https://www.cellsignal.cn/products/primary-antibodies/p44-42-mapk-erk1-2-137f5-rabbit-mab/4695
Anti-p-p38 MAPK(Thr180/Tyr182) Rabbit mAb https://www.cellsignal.cn/products/primary-antibodies/phospho-p38-mapk-thr180-tyr182-d3f9-xp-rabbit-mab/4511

Anti-p38 MAPK Rabbit mAb https://www.cellsignal.cn/products/primary-antibodies/p38-mapk-d13e1-xp-rabbit-mab/8690
Anti-p-AKT (Ser473) Rabbit mAb https://www.cellsignal.cn/products/primary-antibodies/phospho-akt-ser473-d9e-xp-rabbit-mab/4060
Anti-AKT Rabbit mAb https://www.cellsignal.cn/products/primary-antibodies/akt-pan-c67e7-rabbit-mab/4691
Anti-p-NF-κB p65 (Ser536) Rabbit mAb https://www.cellsignal.cn/products/primary-antibodies/phospho-nf-kb-p65-ser536-93h1-rabbit-mab/3033
Anti-NF-κB p65 Rabbit mAb https://www.cellsignal.cn/products/primary-antibodies/nf-kb-p65-d14e12-xp-rabbit-mab/8242
Anti-ANGPTL8 Rabbit pAb https://www.thermofisher.cn/cn/zh/antibody/product/betatrophin-Antibody-Polyclonal/PA5-38043
Anti-ANGPTL8 Rabbit pAb https://www.abcam.cn/products/primary-antibodies/betatrophin-antibody-n-terminal-ab180915.html
Anti-PirB Rat mAb https://www.thermofisher.cn/cn/zh/antibody/product/LILRB3-Antibody-clone-326414-Monoclonal/MA5-24049
Anti-SHP1 Rabbit mAb https://www.cellsignal.cn/products/antibody-conjugates/myc-tag-9b11-mouse-mab-alexa-fluor-555-conjugate/3756
Anti-SHP2 Rabbit mAb https://www.cellsignal.cn/products/primary-antibodies/shp-2-d50f2-rabbit-mab/3397
Anti-phospho-tyrosine https://www.cellsignal.cn/products/primary-antibodies/phospho-tyrosine-mouse-mab-p-tyr-100/9411
Anti-LILRB2 Mouse mAb https://www.rndsystems.com/cn/products/human-lilrb2-cd85d-ilt4-antibody-287219_mab2078
Anti-His Mouse mAb https://www.abcam.cn/products/primary-antibodies/6x-his-tag-antibody-hish8-ab18184.html
Anti-Flag Rat mAb https://www.biolegend.com/en-us/products/purified-anti-dykddddk-tag-antibody-4905
Anti-GST Rabbit https://www.cellsignal.cn/products/primary-antibodies/gst-tag-91g1-rabbit-mab/2625
Anti-TNFα Rabbit pAb https://www.thermofisher.cn/cn/zh/antibody/product/TNF-alpha-Antibody-Polyclonal/AMC3012
Anti-IL-1β Armenian hamster mAb https://www.thermofisher.cn/cn/zh/antibody/product/IL-1-beta-Antibody-clone-B122-Monoclonal/16-7012-81
Anti-IL-6 Rat mAb https://www.thermofisher.cn/cn/zh/antibody/product/IL-6-Antibody-clone-MP5-20F3-Monoclonal/16-7061-81
Anti-F4/80 Rabbit mAb https://www.cellsignal.cn/products/primary-antibodies/f4-80-d2s9r-xp-rabbit-mab/70076
Anti-CD11c Rabbit pAb https://www.servicebio.cn/goodsdetail?id=15496
Anti-CD68 Rabbit pAb https://www.servicebio.cn/goodsdetail?id=17951
Anti-CD11b Rabbit pAb https://www.servicebio.cn/goodsdetail?id=15495
PE anti-mouse F4/80 https://www.biolegend.com/en-us/products/pe-anti-mouse-f4-80-antibody-4068
FITC anti-mouse CD11c https://www.biolegend.com/en-us/products/fitc-anti-mouse-cd11c-antibody-1815
APC anti-mouse CD206 https://www.biolegend.com/en-us/products/apc-anti-mouse-cd206-mmr-antibody-7425
FITC anti-mouse CD45 https://www.biolegend.com/en-us/products/fitc-anti-mouse-cd45-antibody-99
PE anti-mouse CLEC2 https://www.biolegend.com/en-us/products/pe-anti-mouse-clec-2-clec1b-antibody-8759
BV421 anti-mouse F4/80 https://www.biolegend.com/en-us/products/percp-cyanine5-5-anti-mouse-f480-antibody-4303
Pe/Cy7 anti-mouse CD64 https://www.biolegend.com/en-us/products/pe-cyanine7-anti-mouse-cd64-fcgammari-antibody-10062
Percp/Cy5.5 anti-mouse/human CD11b https://www.biolegend.com/en-us/products/percp-cyanine5-5-anti-mouse-human-cd11b-antibody-4257
APC/Cy7 anti-mouse PirB https://www.rndsystems.com/cn/products/mouse-pir-b-alexa-fluor-750-conjugated-antibody-326414_fab2754s
APC anti-mouse Ly6C https://www.biolegend.com/en-us/products/apc-anti-mouse-ly-6c-antibody-6047
PE anti-human CD14 https://www.bdbiosciences.com/zh-cn/products/reagents/flow-cytometry-reagents/research-reagents/single-color-antibodies-ruo/pe-mouse-anti-human-cd14.555398
APC anti-mouse Ly6G/Ly6C https://www.biolegend.com/en-us/products/apc-anti-mouse-ly-6g-ly-6c-gr-1-antibody-456
PerCP anti-mouse CD19 https://www.biolegend.com/en-us/products/percp-anti-mouse-cd19-antibody-4260
APC anti-mouse CD3 https://www.biolegend.com/en-us/products/apc-anti-mouse-cd3-antibody-8055
Anti-β-Actin Mouse mAb https://ptgcn.com/products/Pan-Actin-Antibody-66009-1-Ig.htm
Anti-integrin-α5β1 Rat mAb https://www.sigmaaldrich.cn/CN/zh/product/mm/mab2514
Alexa Fluor-conjugated secondary antibody https://www.servicebio.cn/goodsdetail?id=273
Cy3-conjugated secondary antibody https://www.servicebio.cn/goodsdetail?id=253
Peroxidase AffiniPure goat anti-rabbit-IgG (H+L) https://www.cellsignal.cn/products/secondary-antibodies/anti-rabbit-igg-hrp-linked-antibody/7074
Peroxidase AffiniPure goat anti-rat-IgG (H+L) https://www.cellsignal.cn/products/secondary-antibodies/anti-rat-igg-hrp-linked-antibody/7077
Peroxidase AffiniPure goat anti-mouse-IgG (H+L) https://www.cellsignal.cn/products/secondary-antibodies/anti-mouse-igg-hrp-linked-antibody/7076

Eukaryotic cell lines

Policy information about cell lines and Sex and Gender in Research

Cell line source(s)

RAW264.7 was purchased from Cell Bank of the Chinese Academy of Sciences (catalog number: SCSP-5036); THP-1 was purchased from Cell Bank of the Chinese Academy of Sciences (catalog number: SCSP-567); U-937 was purchased from Cell Bank of the Chinese Academy of Sciences (catalog number: TCHu159); The LX-2 human hepatic stellate cell line was purchased from MERCK (catalog number:SCC064).

Authentication

The cell line was not authenticated

Mycoplasma contamination

All cell lines were tested negative for mycoplasma contamination.

Commonly misidentified lines
(See ICLAC register)

No commonly misidentified cell lines were used in the study.

Animals and other research organisms

Policy information about studies involving animals; ARRIVE guidelines recommended for reporting animal research, and Sex and Gender in Research

Laboratory animals	Wild type C57BL/6 mice<br>hepatocyte-specific Angptl8-knockout mice on a C57BL/6 genetic background, including floxed and Alb-cre.<br>PirB knockout mice on a C57BL/6 genetic background<br>mTmG mice on a C57BL/6 genetic background<br>Mice age: 8-12 weeks; 8 months at the end of CDHFD feeding
Wild animals	The study did not include wild animals.
Reporting on sex	Both sex mice were used for experiments in vitro; Studies regarding nutrition stress predominantly used male mice to establish NASH models due to the the impact of female hormone on metabolism. Female mice also used to verify the key finding.
Field-collected samples	The study did not involve samples collected from the field.
Ethics oversight	All animal protocols were approved by the Institutional Animal Care and Use Committee of the Institute of Model Animals of Tongji Hospital, Huazhong University of Science and Technology (Appl.No.: 068-20220410). The animals received humane care according to the criteria outlined in the Guide for the Care and Use of Laboratory Animals prepared by the National Academy of Sciences and published by the National Institutes of Health.

Note that full information on the approval of the study protocol must also be provided in the manuscript.

Flow Cytometry

Plots

Confirm that:

☒ The axis labels state the marker and fluorochrome used (e.g. CD4-FITC).

☒ The axis scales are clearly visible. Include numbers along axes only for bottom left plot of group (a 'group' is an analysis of identical markers).

☒ All plots are contour plots with outliers or pseudocolor plots.

☒ A numerical value for number of cells or percentage (with statistics) is provided.

Methodology

Sample preparation	The liver of male mice were isolated and washed with D-Hanks' balanced solution for 3-5 min. After blood washed, liver was digested 45 min with collagenase IV. After that, the liver was excised in ice-cold DMEM containing 10% FBS. Cells from digested livers were isolated, suspended in DMEM, filtered through a 100-µm cell strainer, and centrifuged at 50 ×g for 5 min at 4°C. The supernatant, which was enriched in nonparenchymal cells, was loaded on a double-layer discontinuous iodixanol gradient of 11.5% and 20% OptiPrep (Sigma, MO, USA, D1556) and centrifuged at 1400 ×g for 17 min at 4°C without applying the centrifuge brake. The upper layer of 20% OptiPrep was collected and allowed to attach to cell culture plates in DMEM with 10% FBS.
Instrument	CytoFLEX (Beckman Coulter); 3 laster, 13 channel; MACSQuantTM (Miltenyi Biotec, Auburn, CA, USA)
Software	FlowJo version: v7.6.5 for flow cytometry; BD FACSDiva software was used for cell sorting.
Cell population abundance	Sort purity was routinely over 95% on post-sort checks
Gating strategy	Cells were gated by FSC/SSC gates and then FSC/FSC-width to select single cells. Cells were treated with Zombie Aqua Fixable Viability Kit (BioLegend, CA, USA, #B297827) to discriminate live and dead cells. After that the detail gating strategy was showed as follows:<br>Mouse hepatic macrophages: CD45+, F4/80+<br>Mouse Kupffer cells: CD45+, F4/80hi, CD11blo, CLEC2hi, Ly6c-<br>Mouse MDMs: CD45+, CD64+ F4/80lo, CD11bhi, CLEC2lo, Ly6c+<br>Mouse Ly6c+ monocytes: CD45+, F4/80lo, CD11blo, CLEC2lo, Ly6c+<br>M1 macrophages: F4/80+, CD11c+, CD206-<br>M2 macrophages: F4/80+, CD11c-, CD206+<br>The gating strategy was provided in Supplementary Figures.

☒ Tick this box to confirm that a figure exemplifying the gating strategy is provided in the Supplementary Information.
